# Supplementary material for: Fitness translocation: improving variant effect prediction with biologically-grounded data augmentation
Source: Bioinformatics. 2026 Jun 20;42(7):btag415. doi: 10.1093/bioinformatics/btag415 (PMC13360282; doi:10.1093/bioinformatics/btag415)
Supplement: btag415_Supplementary_Data [file btag415_supplementary_data.zip › supplemental.pdf]

Supplemental S1: Protein Identity, Similarity, Fitness, and mutations.

Table S1.1: Summary of the dataset. The pairwise sequence similarities are computed using EMBOSS Needle and EMBOSS Water [16], respectively for global and local alignment.

**Protein Family**	**Homolog**	**Number of Variants**	**EMBOSS Needle**			**EMBOSS Water**		
			SsIGPS	TmIGPS	TtIGPS	SsIGPS	TmIGPS	TtIGPS
IGPS	SsIGPS	1497	100%	56.7%	55.8%	100%	58.8%	60.1%
	TmIGPS	1502	56.7%	100%	49.1%	58.8%	100%	51.2%
	TtIGPS	1489	55.8%	49.1%	100%	60.1%	51.2%	100%
			amacGFP	cgreGFP	ppluGFP	amacGFP	cgreGFP	ppluGFP
GFP	amacGFP	35500	100%	62.6%	29.9%	100%	65.9%	41.5%
	cgreGFP	26165	62.6%	100%	32.3%	65.9%	100%	38.4%
	ppluGFP	32260	29.9%	32.3%	100%	41.5%	38.4%	100%
			XBB.1.5	BA.2	–	XBB.1.5	BA.2	–
SARS-CoV-2 Spike	XBB.1.5	7029	100%	98.8%	–	100%	98.9%	–
	BA.2	7129	98.9%	100%	–	98.9%	100%	–

Table S1.2: Summary of the dataset. Fitness range and mean pm SD for each homolog alongside their corresponding assay.

Protein	Homolog	Assay	Fitness Range	Mean ± SD
IGPS	SsIGPS	Enzymatic Activity	-3.392 – 1.239	-0.549 ± 0.611
	TmIGPS	Enzymatic Activity	-2.157 – 1.951	-0.687 ± 0.608
	TtIGPS	Enzymatic Activity	-2.223 – 0.862	-0.461 ± 0.608
GFP	amacGFP	Fluorescence	-1.318 – 0.301	-0.320 ± 0.454
	cgreGFP	Fluorescence	-1.726 – 0.106	-0.850 ± 0.770
	ppluGFP	Fluorescence	-1.455 – 0.219	-0.355 ± 0.517
Sars-CoV-2 spike	XBB.1.5	Cell Entry	-6.862 – 0.340	-1.406 ± 2.066
	BA.2	Cell Entry	-6.619 – 0.175	-0.895 ± 1.792
	XBB.1.5	ACE2 Binding	-3.726 – 4.013	0.036 ± 0.525
	BA.2	ACE2 Binding	-7.446 – 5.283	0.238 ± 0.867

Table S1.3: Summary of the dataset. Sequence and mutation informations for each homolog.

Protein	Homolog	Num. of Variants	Seq. lenght	Num. of Mut. Sites	Coverage Type	Num. of Mutations
IGPS	SsIGPS	1497	222	80	Saturation	20 AA/site
	TmIGPS	1502	221	80	Saturation	20 AA/site
	TtIGPS	1489	220	80	Saturation	20 AA/site
GFP	amacGFP	35500	244	237	Error-prone PCR	avg 3/variant
	cgreGFP	26165	235	235	Error-prone PCR	avg 3/variant
	ppluGFP	32260	222	221	Error-prone PCR	avg 3/variant
SARS-CoV-2 spike	XBB.1.5	7029	1253	1234	Targeted	avg 2/variant
	BA.2	7129	1253	1240	Targeted	avg 2/variant
	XBB.1.5	7029	1253	1149	Targeted	avg 2/variant
	BA.2	7129	1253	1196	Targeted	avg 2/variant

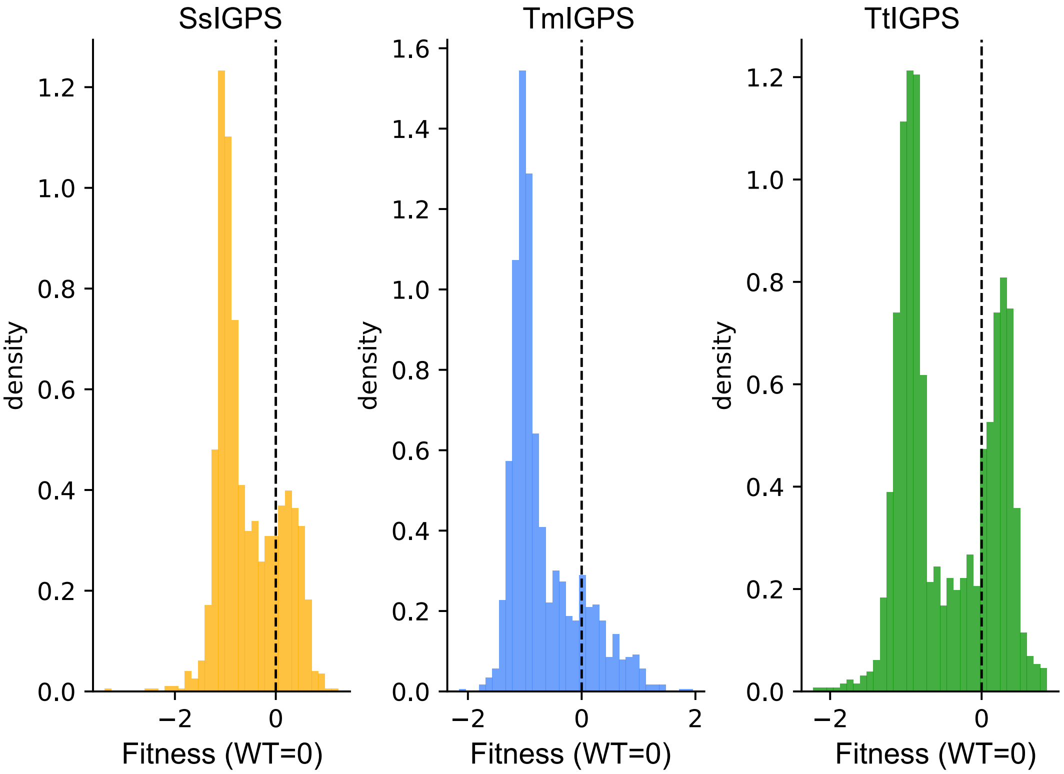

Figure S1.1: IGPS orthologs fitness distributions. The dotted vertical line is the WT=0 fitness

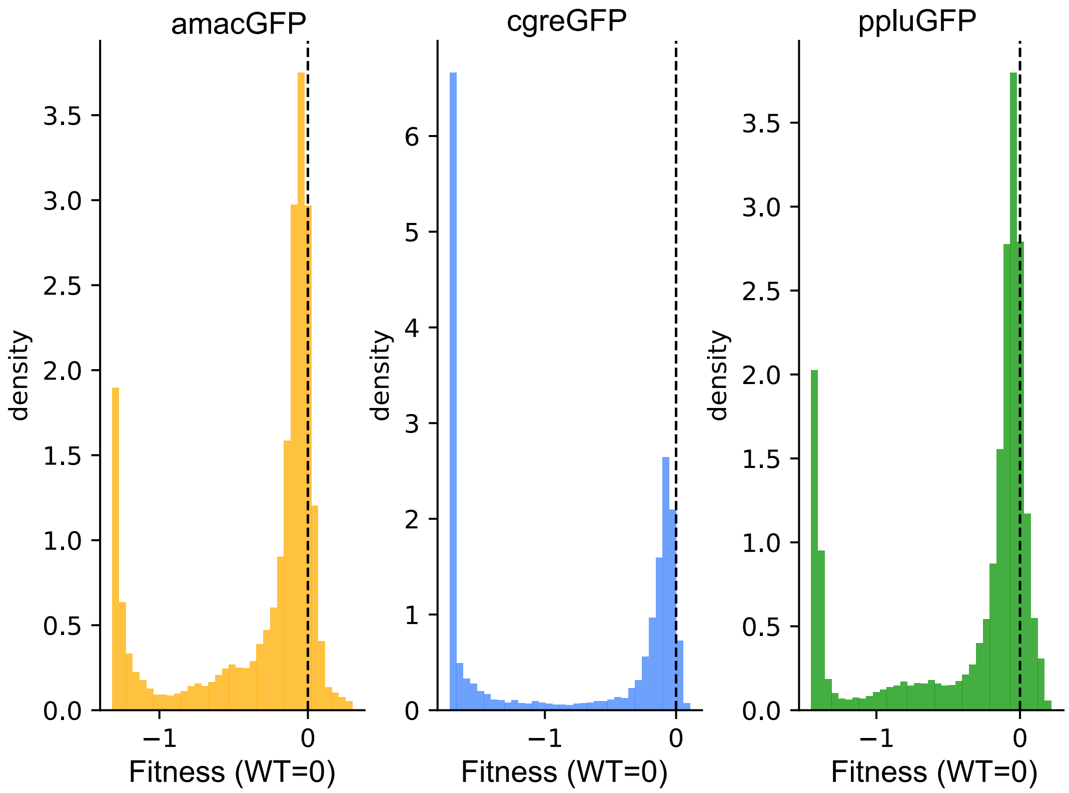

Figure S1.2: GFP orthologs fitness distributions. The dotted vertical line is the WT=0 fitness

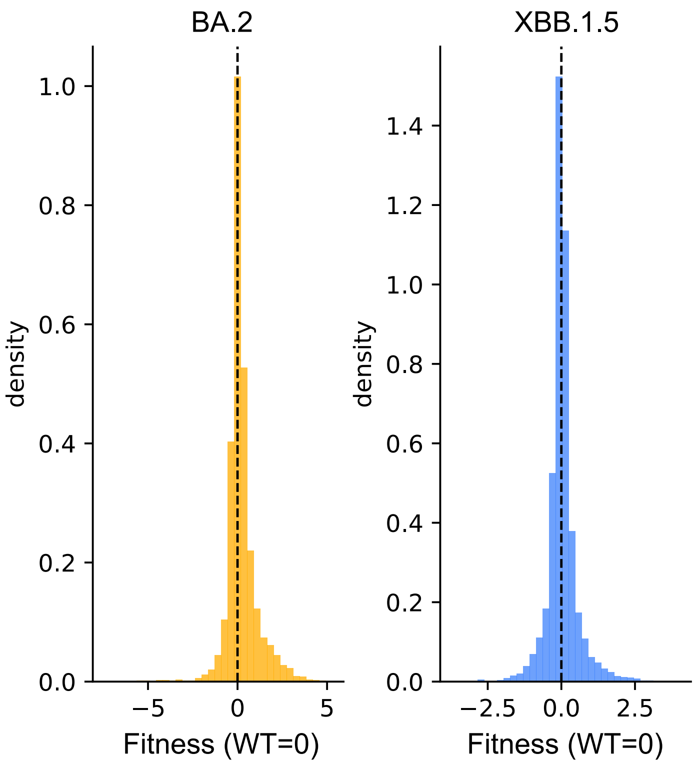

Figure S1.3: SARS-CoV-2 orthologs (ACE2 binding assay) fitness distributions. The dotted vertical line is the WT=0 fitness

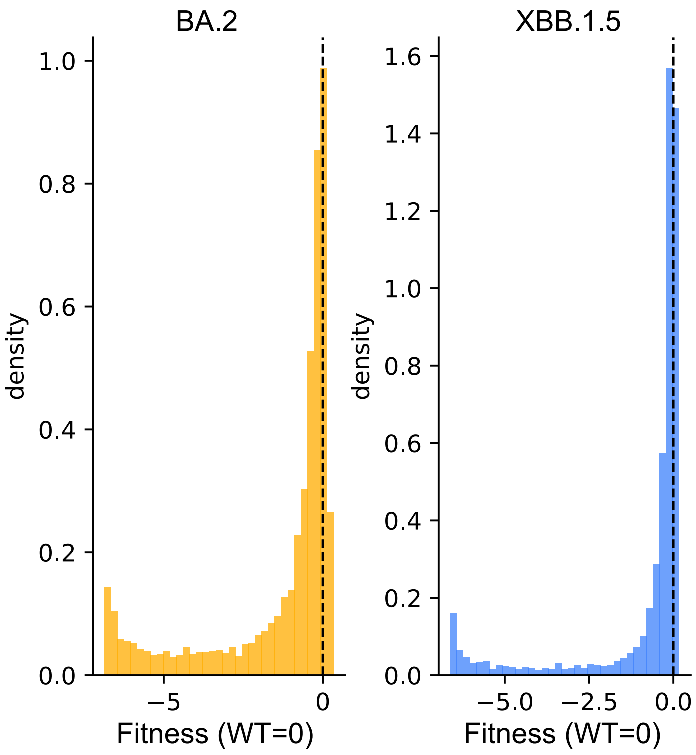

Figure S1.4: SARS-CoV-2 orthologs Spike (cell entry assay) fitness distributions. The dotted vertical line is the WT=0 fitness

Supplemental S2: homolog selection algorithms

Algorithm 1 homolog selection algorithm

```
Require: target, homologs, N, α
  differences ← []
  significant ← []

  for n ← 1 to N do                                          ▷ Compute paired differences
      train, validation ← split_dataset(target)
      t_result ← evaluate_model(train, validation)
      for all hom ∈ homologs do
          trans ← translocate(target, hom)
          result ← evaluate_model(train||trans, validation)
          append (hom, result − t_results) to differences
      end for
  end for

  for all hom ∈ homologs do                                  ▷ Compute differences significance
      σ_e ← standard_error(differences, hom)
      Δμ ← mean_difference(differences, hom)
      if p-value(Δμ, σ_e, N) < α then
          append (hom, Δμ) to significant
      end if
  end for

  hom_best, Δμ_best ← extract_best(significant)
  significant ← remove_best(significant)

  candidate_homs ← sort_by_Δμ(significant)
  optimal_set ← hom_best

  for all hom ∈ candidate_homs do                            ▷ Sort out candidate combinations
      new_results ← []
      for n ← 1 to N do
          trans ← translocate(target, hom, optimal_set)
          result ← evaluate_model(train||trans, validation)
          append result to new_results
      end for

      Δμ_new ← mean(new_results)

      if Δμ_new > Δμ_best then
          Δμ_best ← Δμ_new
          add hom to optimal_set
      end if
  end for

  return optimal_set
```

Supplemental S3: Original and Translocated Embedding Comparison

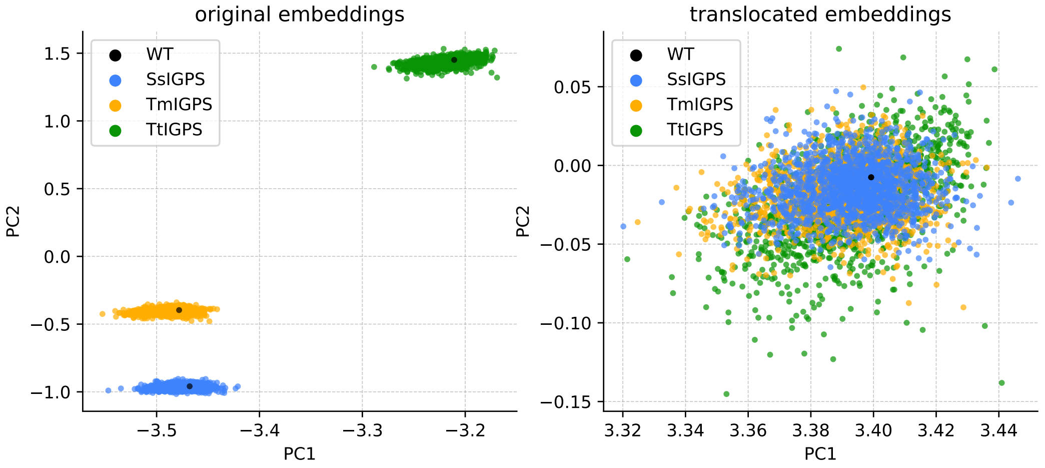

Figure S3.1: Principal Component Analysis (PCA) of IGPS protein embeddings, before and after fitness translocation. Variant embeddings from different homologs are initially separated in embedding space but evenly aggregated after fitness translocation, reflecting the transfer of mutational effects to the target protein.

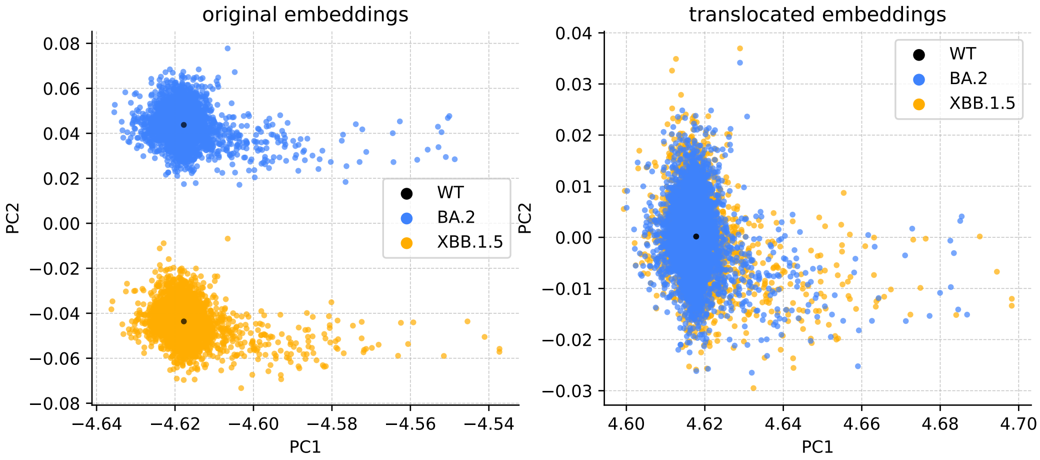

Figure S3.2: Principal Component Analysis (PCA) of SARS-CoV-2 Spike protein embeddings, before and after fitness translocation. Variant embeddings from different homologs are initially separated in embedding space but evenly aggregated after fitness translocation, reflecting the transfer of mutational effects to the target protein.

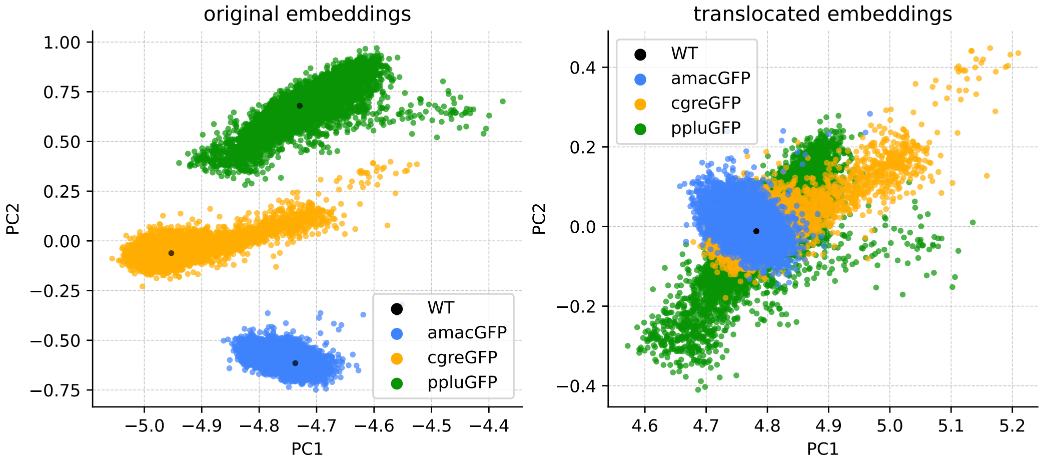

Figure S3.3: Principal Component Analysis (PCA) of GFP protein embeddings, before and after fitness translocation. Variant embeddings from different homologs are initially separated in embedding space but partially aggregated after fitness translocation, reflecting the transfer of mutational effects to the target protein.

Supplemental S4: Embeddings Generation Computational Cost

Table S4.1: Computational cost to obtain sequence embeddings for each protein family. The time/sequence increase with sequence length and dataset length. The longest dataset to process was GFP sequences and took less 35 minutes to obtain with one standard NVIDIA A6000 GPU and around 13h00 with a CPU.

**Protein Family**	**Total seq**	**Seq lenght**	**GPU**		**CPU**	
			Full time (s)	time/seq (s)	Full time (s)	time/seq (s)
IGPS	4488	221	99	0.022	2226	0.496
GFP	93925	234	2066	0.022	47056	0.501
SARS-CoV-2 Spike	14158	1253	1741	0.123	40506	2.861

Supplemental S5: Cross-validation Strategies Comparison - Random Splitting vs Grouped β-Strand Splitting.

Table S5.1: IGPS fitness translocation results. Comparison between random splitting cross-validation and grouped β-strands splitting cross-validation.

			Target training sizes						
**Grouping**	**Target**	**Homolog**	45	75	108	148	196	252	318
random	SsIGPS		0.404	0.472	0.534	0.578	0.610	0.642	0.667
	SsIGPS	TtIGPS	0.608	0.625	0.639	0.657	0.671	0.686	0.702
β-strands	SsIGPS		0.395	0.474	0.529	0.572	0.614	0.645	0.663
	SsIGPS	TtIGPS	0.518	0.551	0.587	0.607	0.635	0.659	0.674
random	TmIGPS		0.227	0.366	0.430	0.496	0.533	0.568	0.601
	TmIGPS	SsIGPS	0.489	0.518	0.548	0.577	0.595	0.617	0.640
β-strands	TmIGPS		0.221	0.365	0.425	0.490	0.528	0.563	0.597
	TmIGPS	SsIGPS	0.414	0.448	0.489	0.542	0.567	0.597	0.615
random	TtIGPS		0.416	0.487	0.546	0.591	0.623	0.661	0.690
	TtIGPS	SsIGPS	0.569	0.593	0.625	0.650	0.673	0.697	0.721
β-strands	TtIGPS		0.411	0.492	0.542	0.590	0.623	0.658	0.687
	TtIGPS	SsIGPS	0.471	0.527	0.576	0.607	0.639	0.673	0.699

Supplemental S6: SARS-CoV-2 Spike protein Cell Entry, ESM-1v, SVR - Lasso - RF, No-Selection

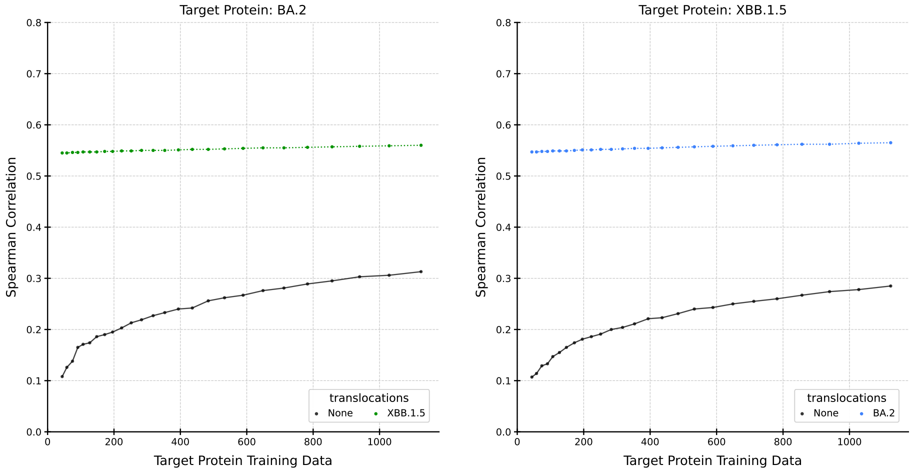

Figure S6.1: SARS-CoV-2 spike protein, ESM-1v pLM, and SVR predictor.

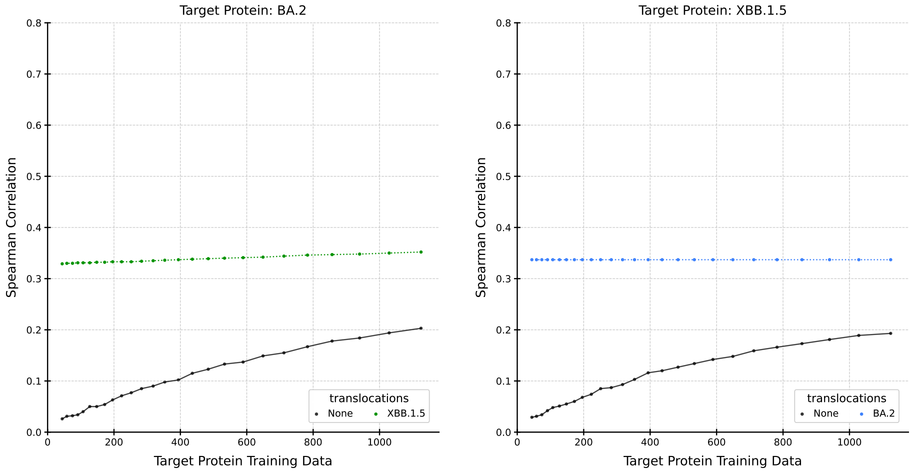

Figure S6.2: SARS-CoV-2 spike protein, ESM-1v pLM, and Lasso predictor.

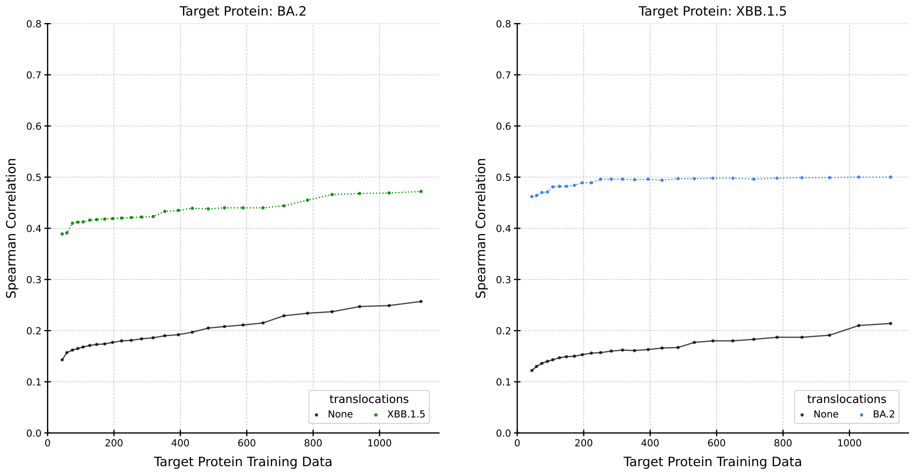

Figure S6.3: SARS-CoV-2 spike protein, ESM-1v pLM, and RF predictor.

Supplemental S7: SARS-CoV-2 Spike protein ACE2 Bindings, ESM-1v, SVR - Lasso - RF, No-Selection

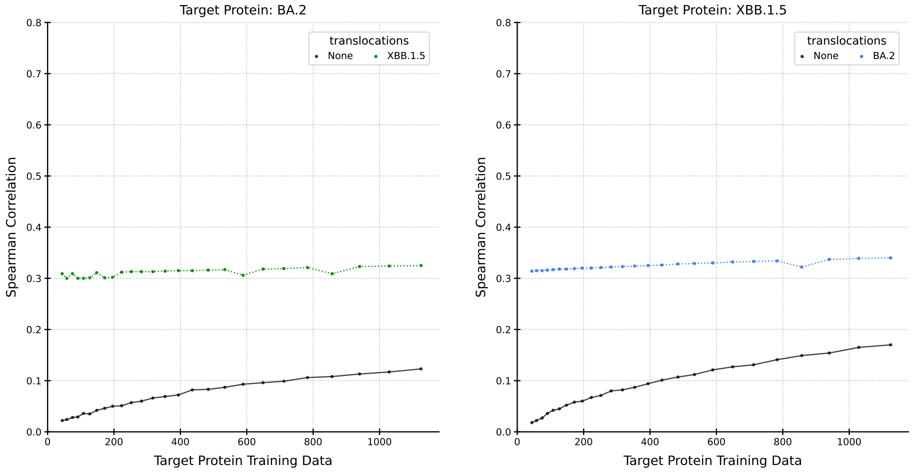

Figure S7.1: SARS-CoV-2 spike protein ACE2 Binding, ESM-1v pLM, and SVR predictor.

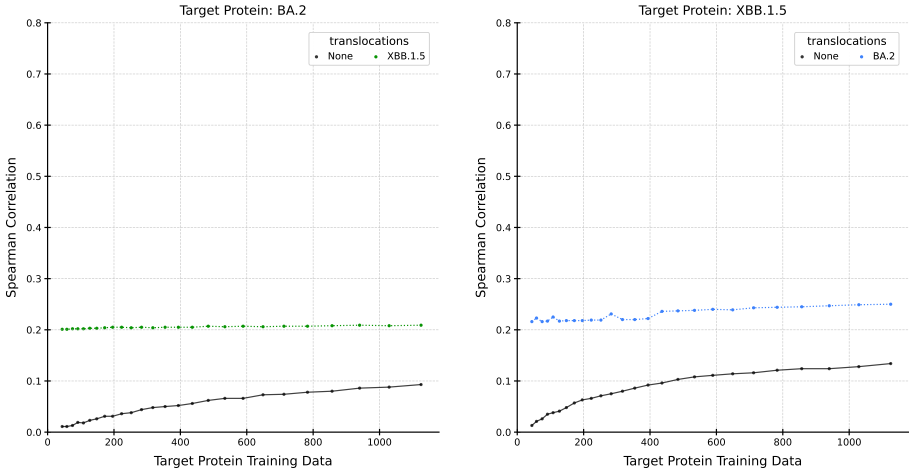

Figure S7.2: SARS-CoV-2 spike protein ACE2 Binding, ESM-1v pLM, and Lasso predictor.

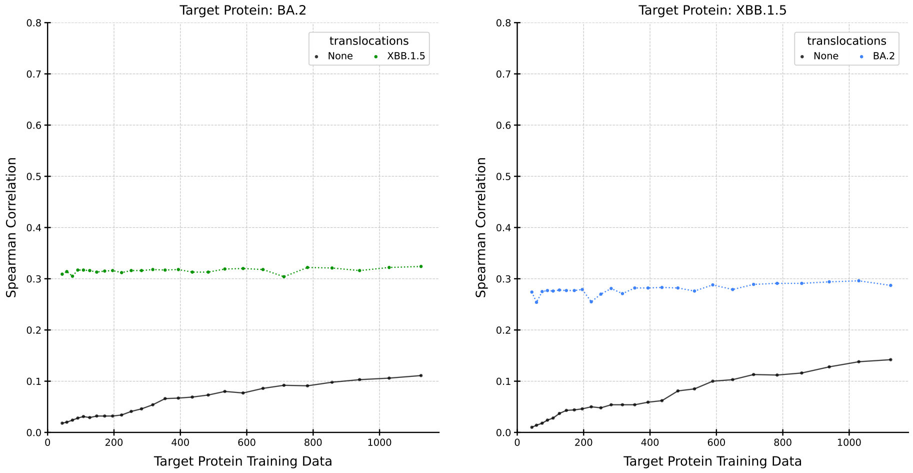

Figure S7.3: SARS-CoV-2 spike protein ACE2 Binding, ESM-1v pLM, and RF predictor.

Supplemental S8: IGPS, ESM-1v, SVR - Lasso - RF, No-Selection

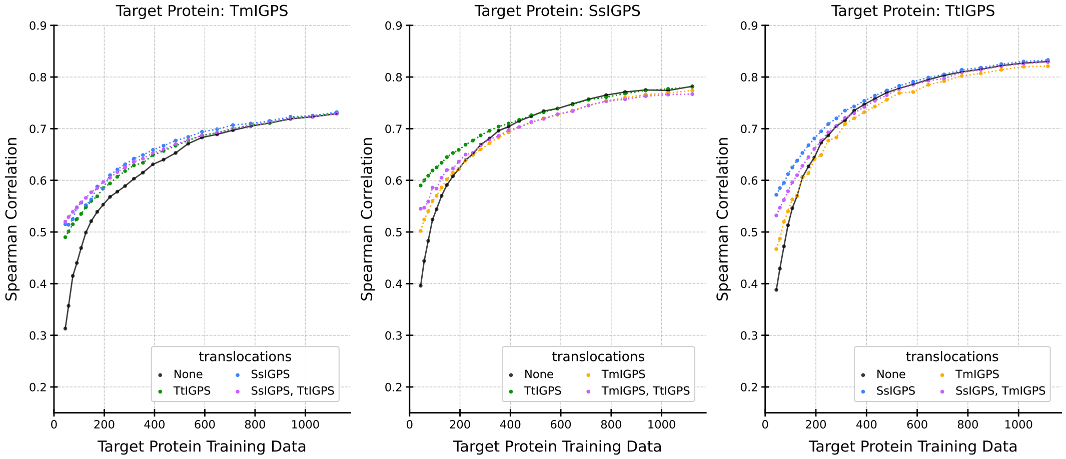

Figure S8.1: IGPS orthologs, ESM-1v pLM, and SVR predictor.

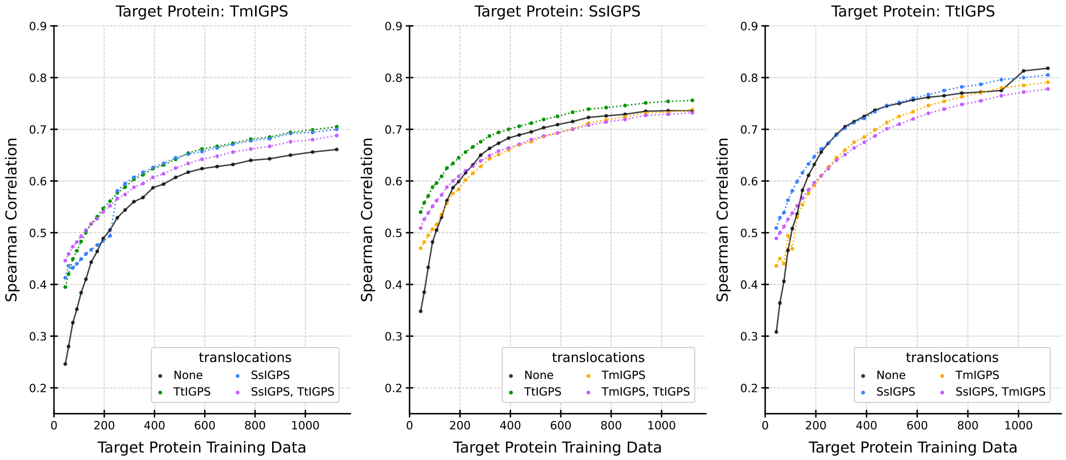

Figure S8.2: IGPS orthologs, ESM-1v pLM, and lasso predictor.

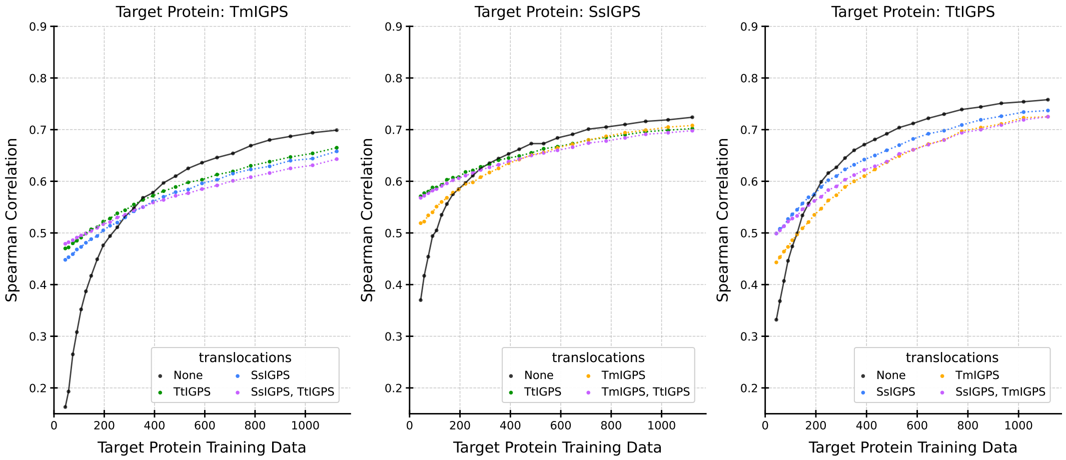

Figure S8.3: IGPS orthologs, ESM-1v pLM, and RF predictor.

Supplemental S9: GFP, ESM-1v, SVR - Lasso - RF, No-Selection

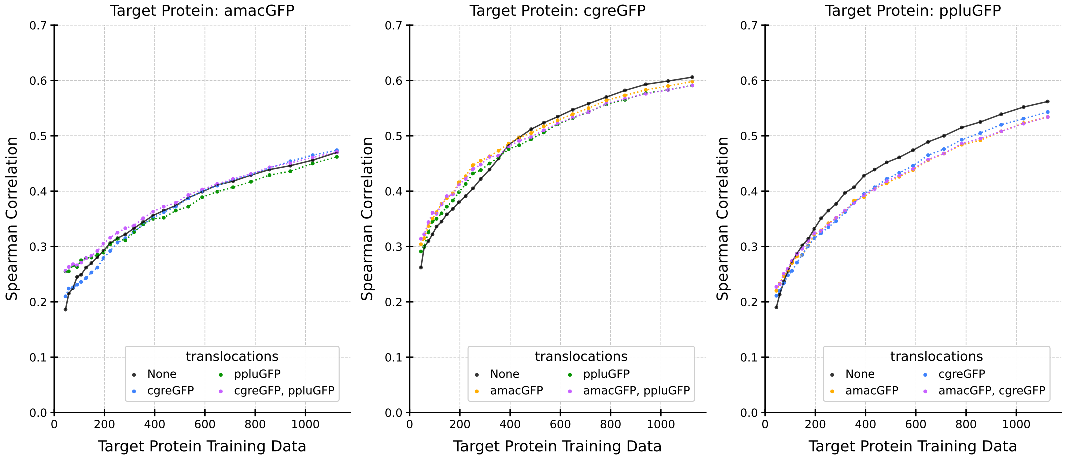

Figure S9.1: GFP orthologs, ESM-1v pLM, and SVR predictor.

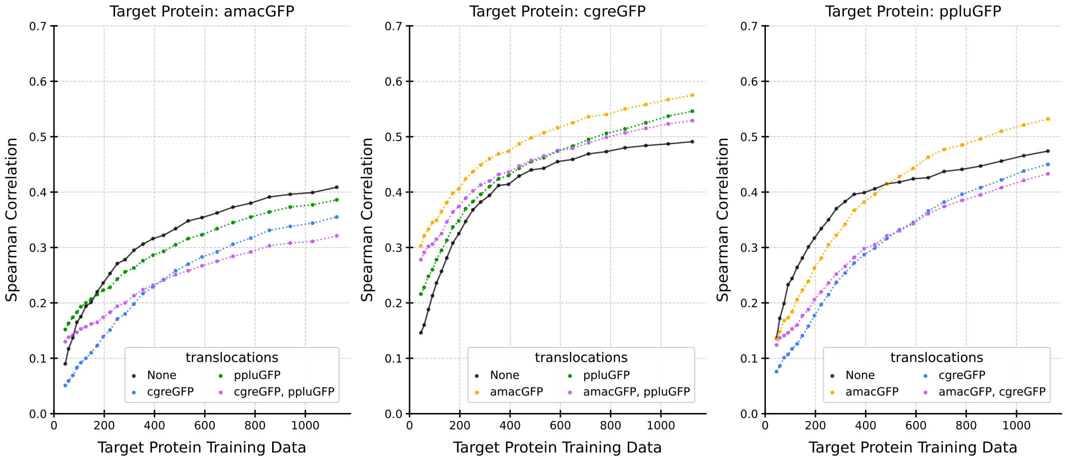

Figure S9.2: GFP orthologs, ESM-1v pLM, and Lasso predictor.

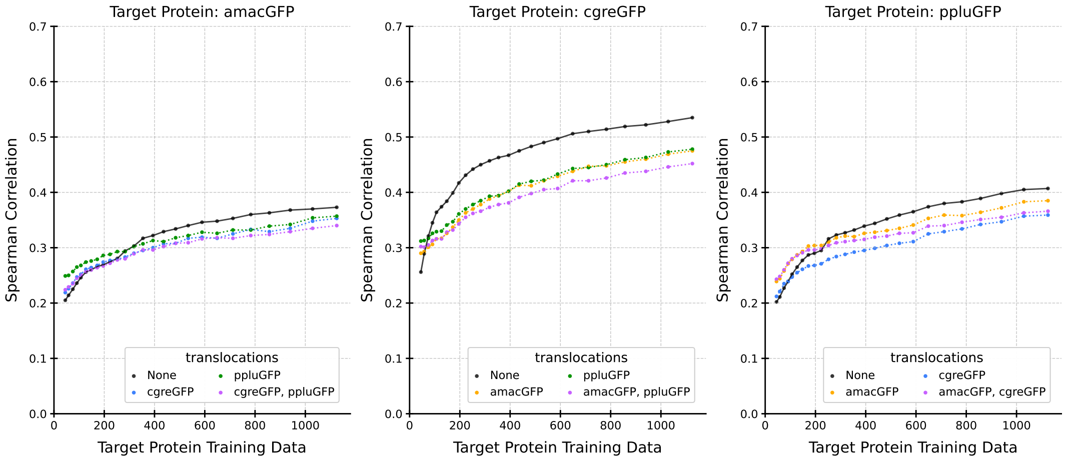

Figure S9.3: GFP orthologs, ESM-1v pLM, and RF predictor.

Supplemental S10: SARS-CoV-2 Spike protein Cell Entry, ESM2, Lasso - SVR - RF, No-Selection

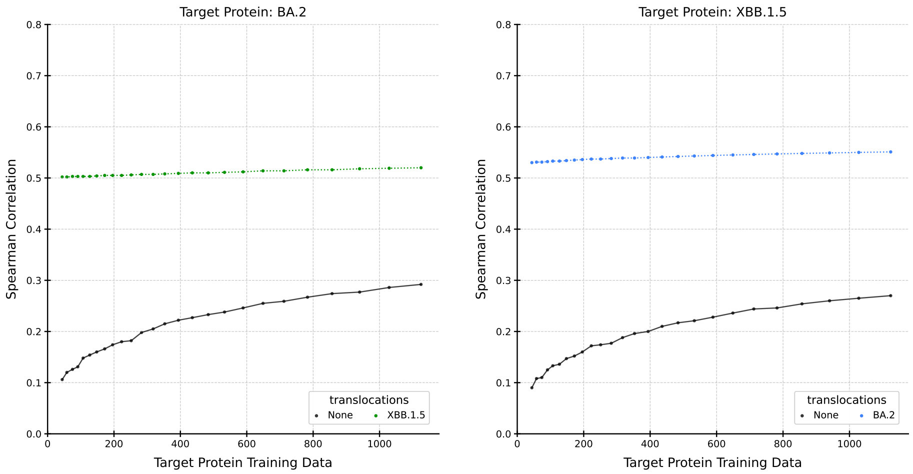

Figure S10.1: SARS-CoV-2 spike protein Cell Entry, ESM2 pLM, and SVR predictor.

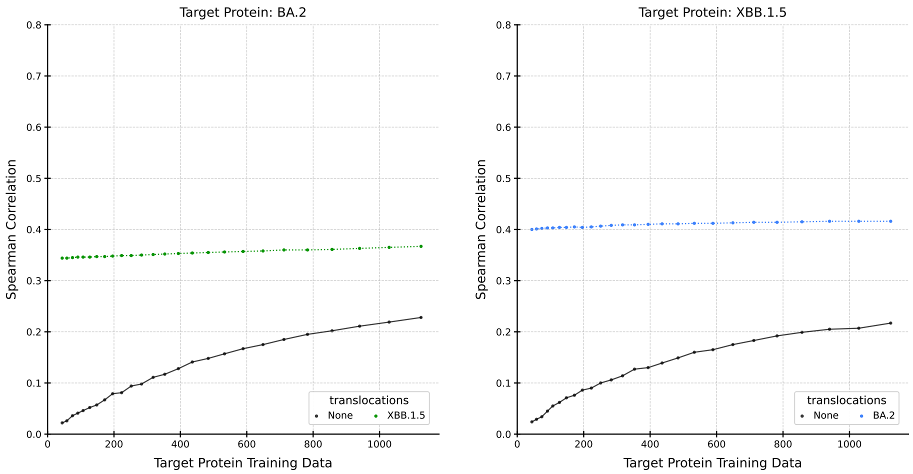

Figure S10.2: SARS-CoV-2 spike protein Cell Entry, ESM2 pLM, and Lasso predictor.

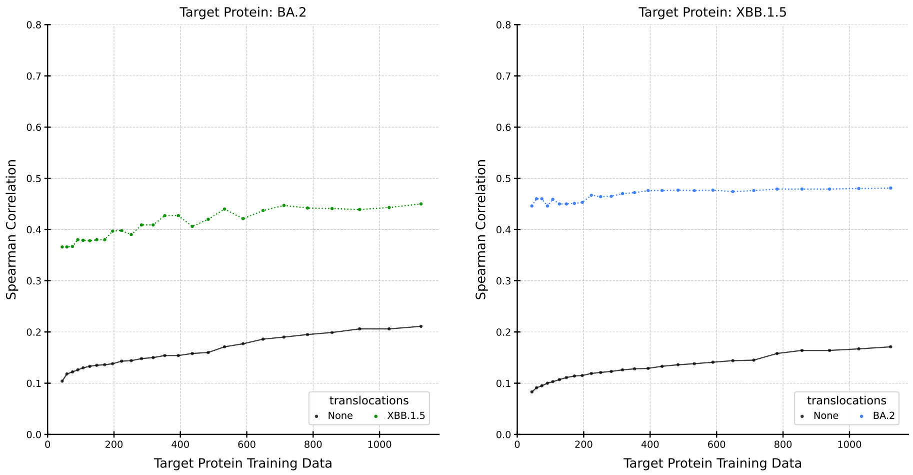

Figure S10.3: SARS-CoV-2 spike protein Cell Entry, ESM2 pLM, and RF predictor.

Supplemental S11: SARS-CoV-2 Spike protein ACE2 Binding, ESM2, Lasso - SVR - RF, No-Selection

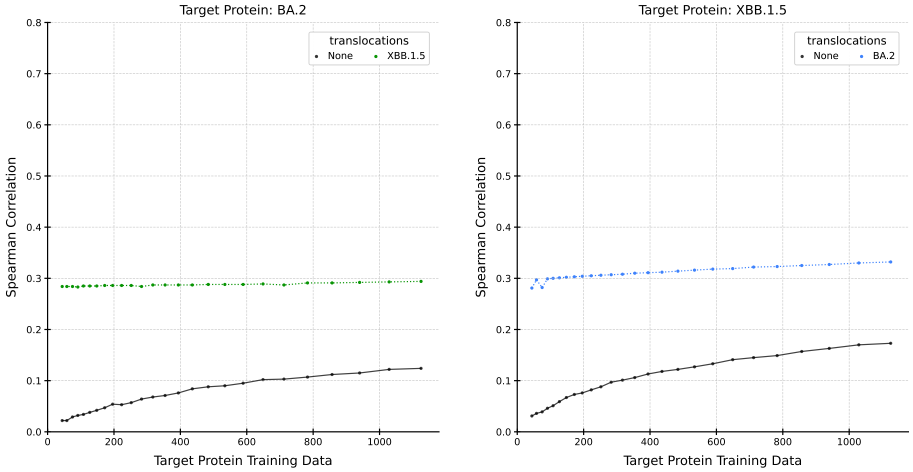

Figure S11.1: SARS-CoV-2 spike protein ACE2 Binding, ESM2 pLM, and SVR predictor.

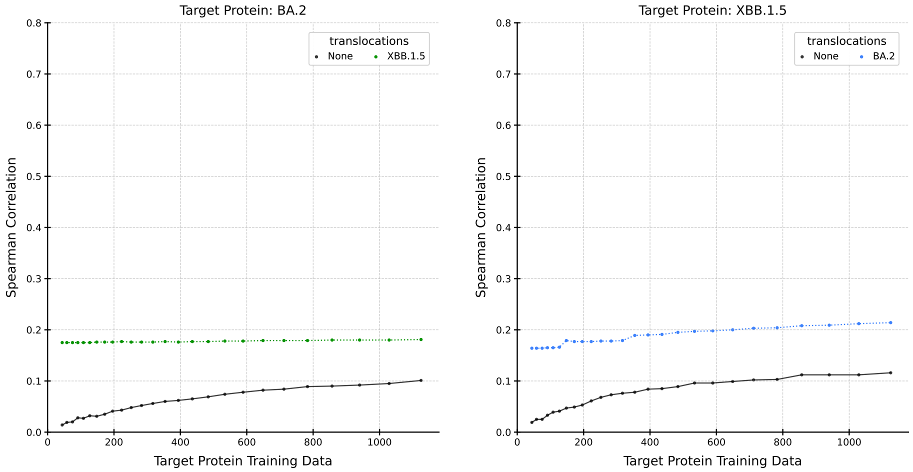

Figure S11.2: SARS-CoV-2 spike protein ACE2 Binding, ESM2 pLM, and Lasso predictor.

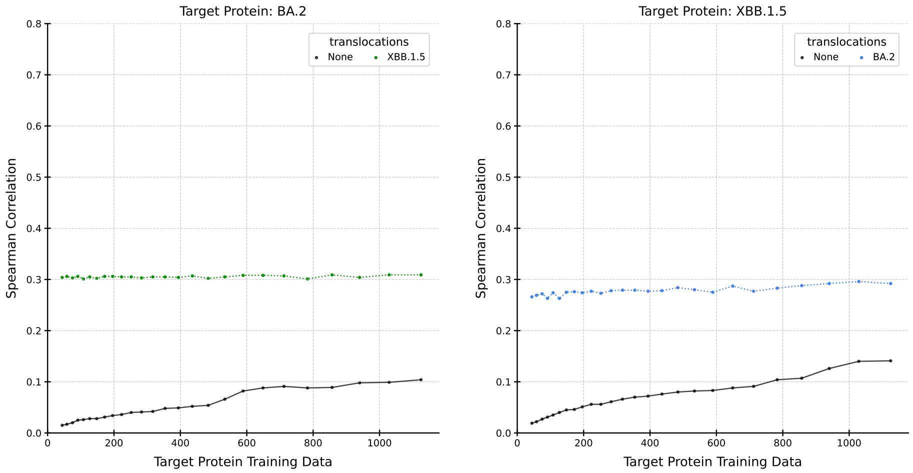

Figure S11.3: SARS-CoV-2 spike protein ACE2 Binding, ESM2 pLM, and RF predictor.

Supplemental S12: IGPS, ESM2, Lasso - SVR - RF, No-Selection

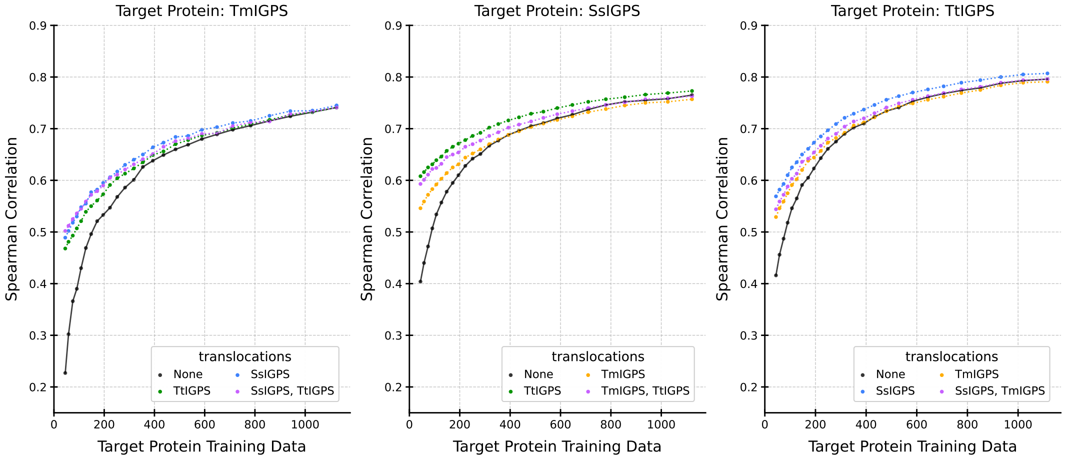

Figure S12.1: IGPS, ESM2 pLM, and SVR predictor.

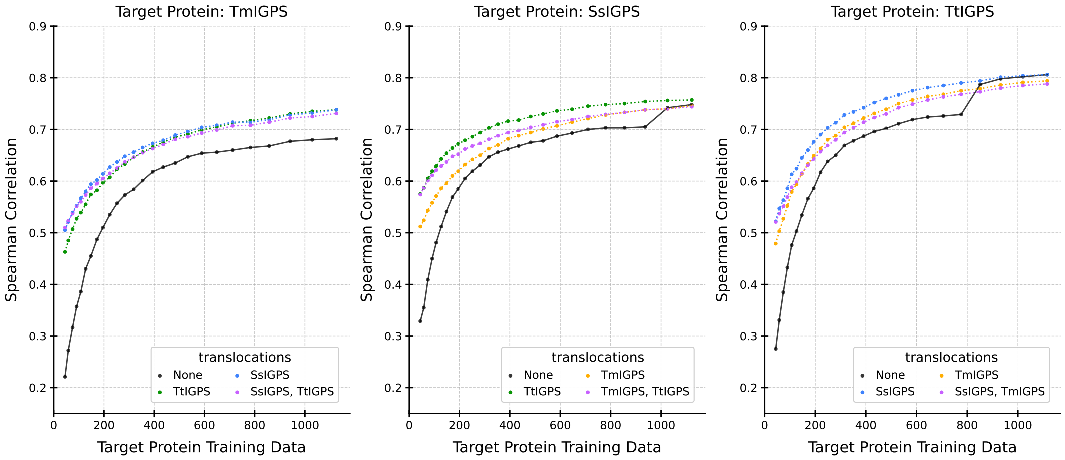

Figure S12.2: IGPS, ESM2 pLM, and Lasso predictor.

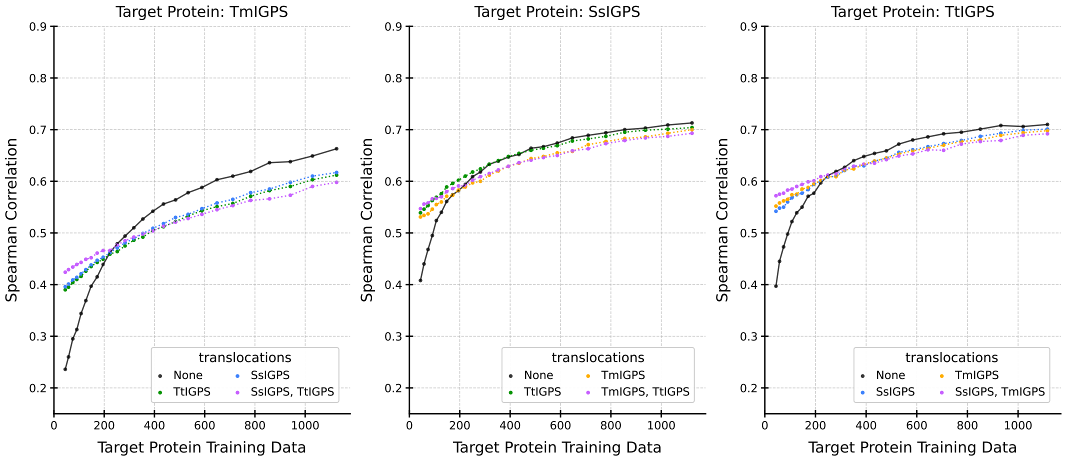

Figure S12.3: IGPS, ESM2 pLM, and RF predictor.

Supplemental S13: GFP, ESM2, SVR - Lasso - RF, No-Selection

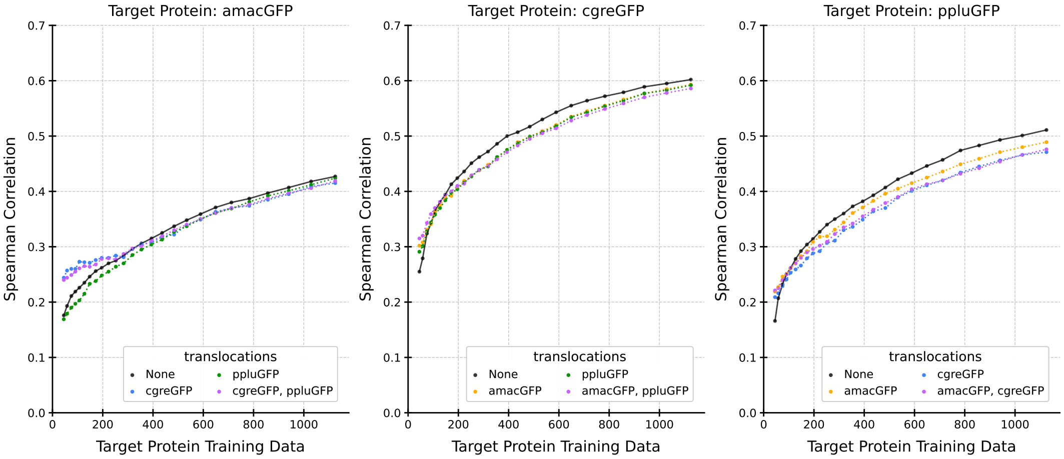

Figure S13.1: GFP, ESM2 pLM, and SVR predictor.

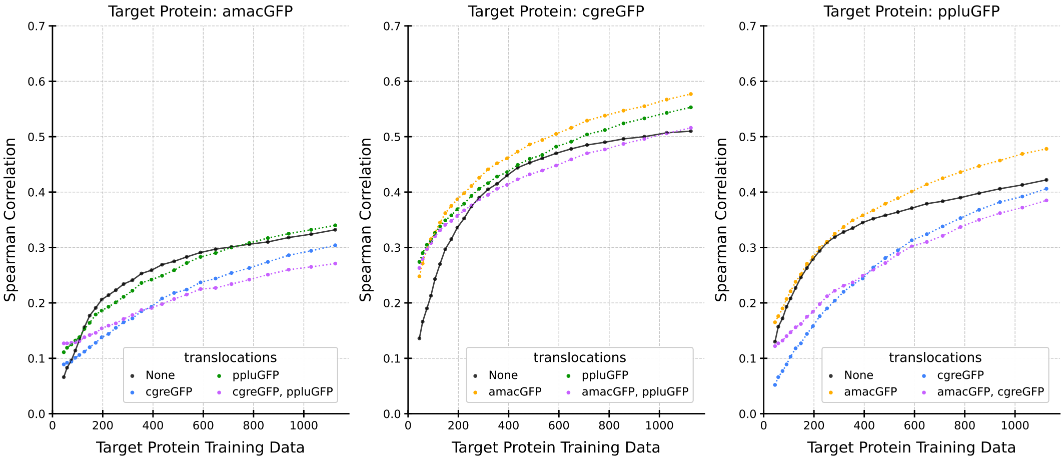

Figure S13.2: GFP, ESM2 pLM, and Lasso predictor.

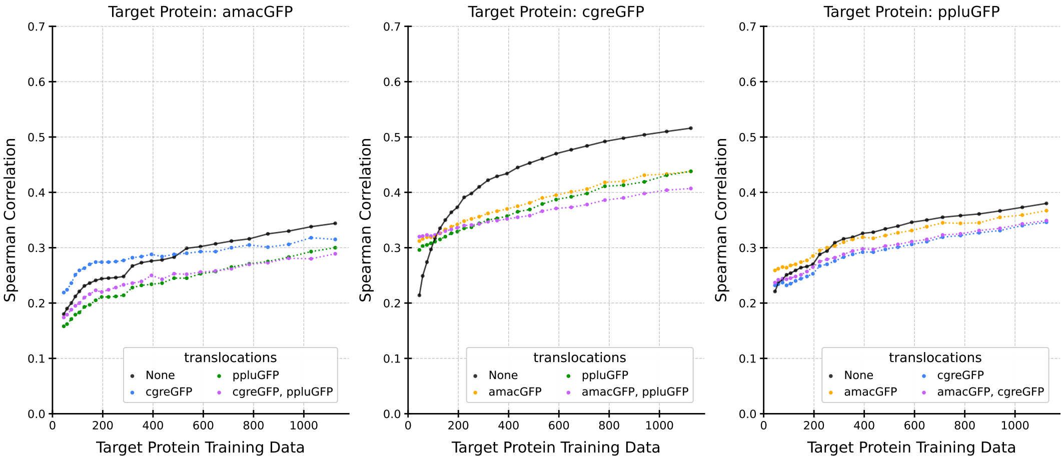

Figure S13.3: GFP, ESM2 pLM, and RF predictor.

Supplemental S14: SARS-CoV-2 Spike protein Cell Entry, ESM-1v, SVR - Lasso - RF, Statistical-Greedy

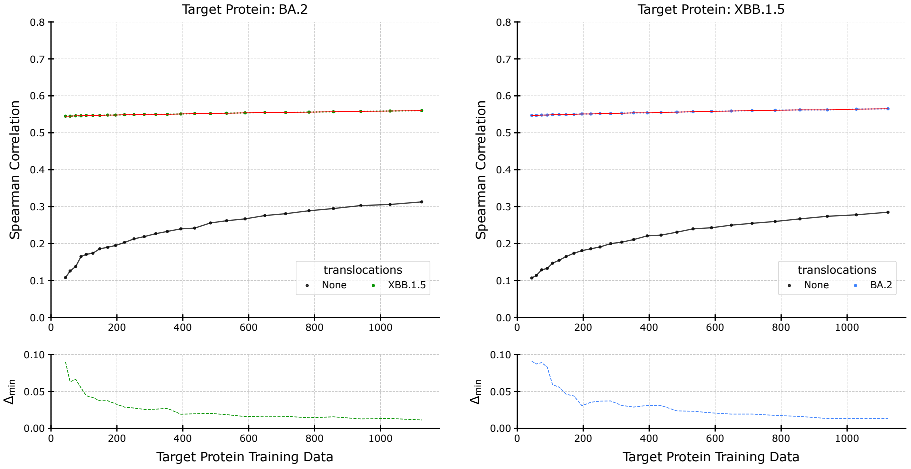

Figure S14.1: SARS-CoV-2 spike protein, ESM-1v pLM, and SVR predictor.

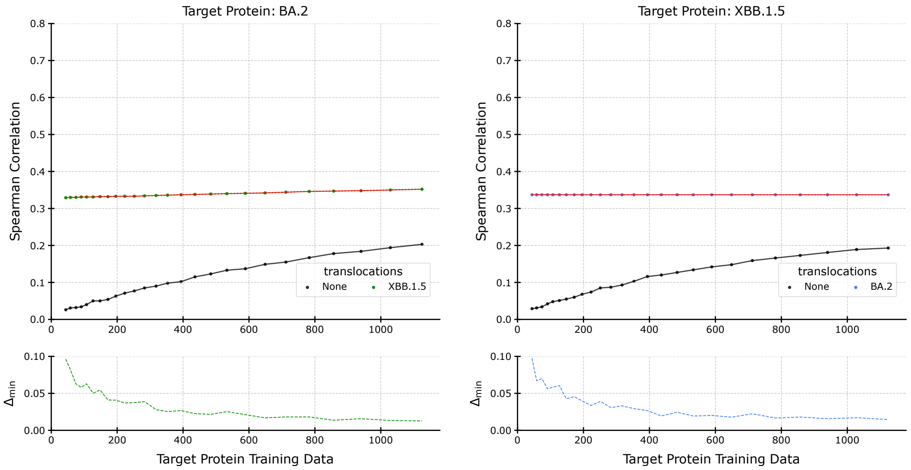

Figure S14.2: SARS-CoV-2 spike protein, ESM-1v pLM, and Lasso predictor.

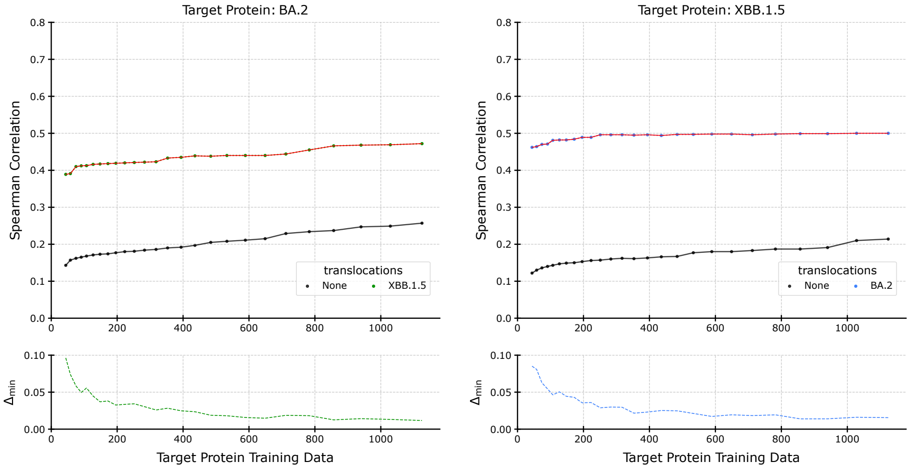

Figure S14.3: SARS-CoV-2 spike protein, ESM-1v pLM, and RF predictor.

Supplemental S15: SARS-CoV-2 Spike protein ACE2 Binding, ESM-1v, SVR - Lasso - RF, Statistical-Greedy

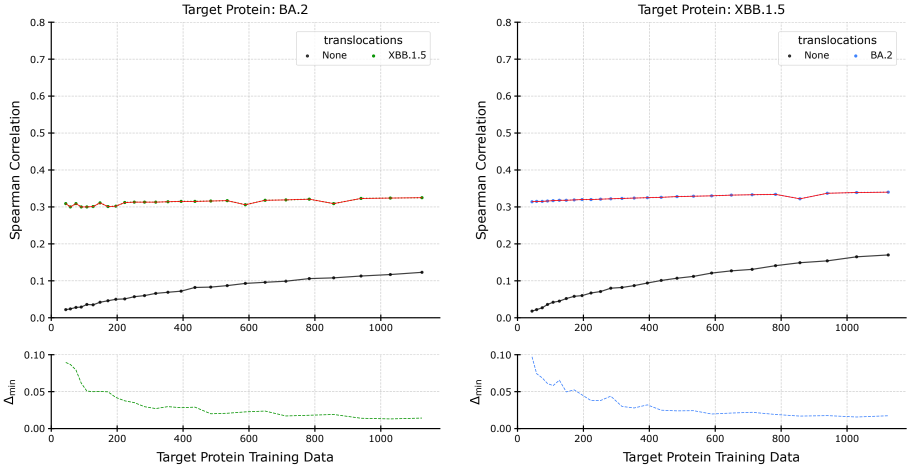

Figure S15.1: SARS-CoV-2 spike protein ACE2 Binding, ESM-1v pLM, and SVR predictor.

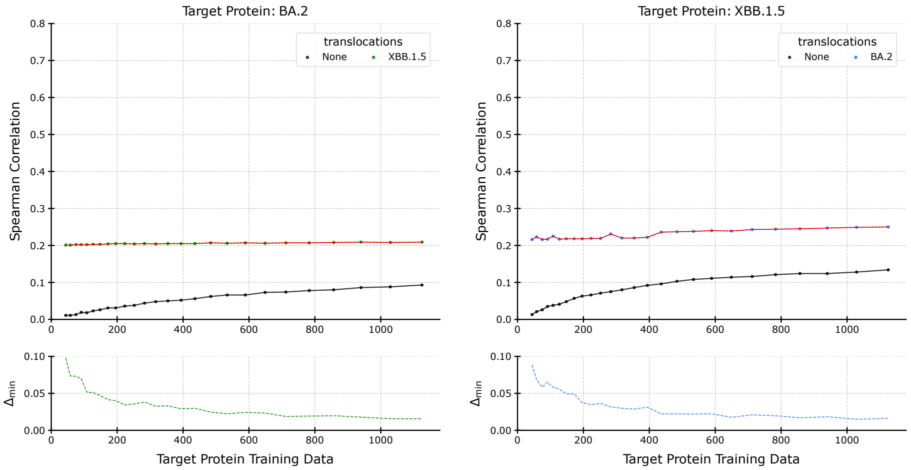

Figure S15.2: SARS-CoV-2 spike protein ACE2 Binding, ESM-1v pLM, and Lasso predictor.

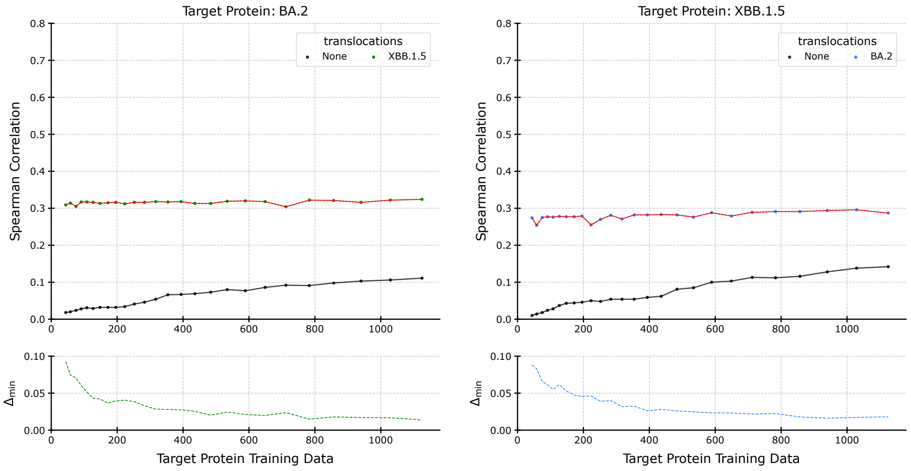

Figure S15.3: SARS-CoV-2 spike protein ACE2 Binding, ESM-1v pLM, and RF predictor.

Supplemental S16: IGPS, ESM-1v, SVR - Lasso - RF, Statistical-Greedy

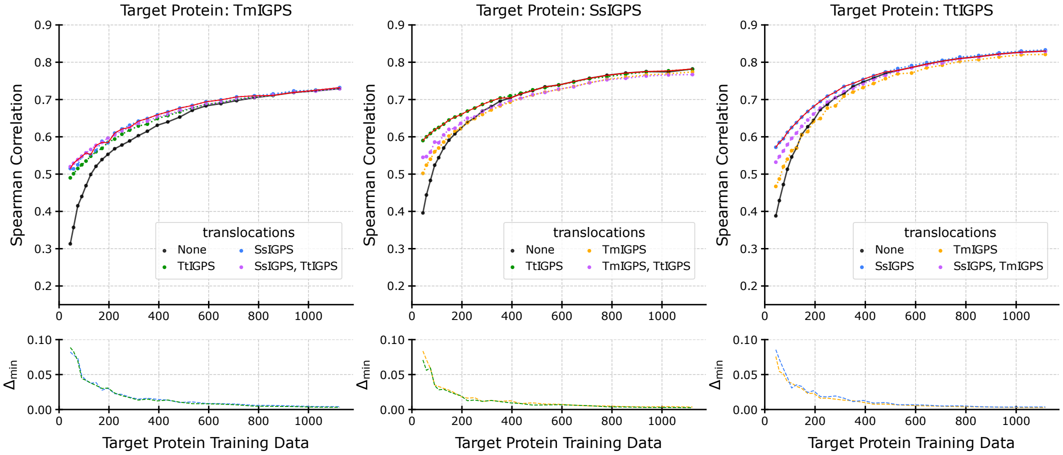

Figure S16.1: IGPS, ESM-1v pLM, and SVR predictor.

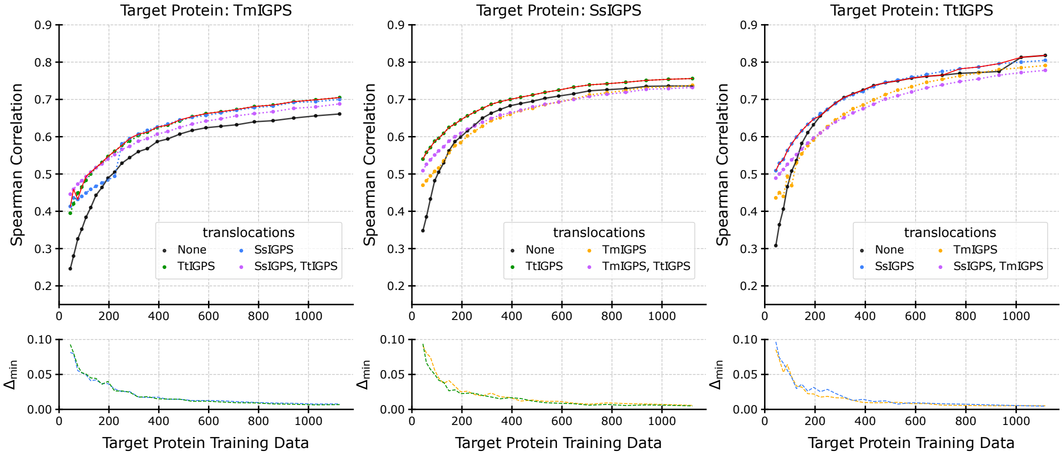

Figure S16.2: IGPS, ESM-1v pLM, and Lasso predictor.

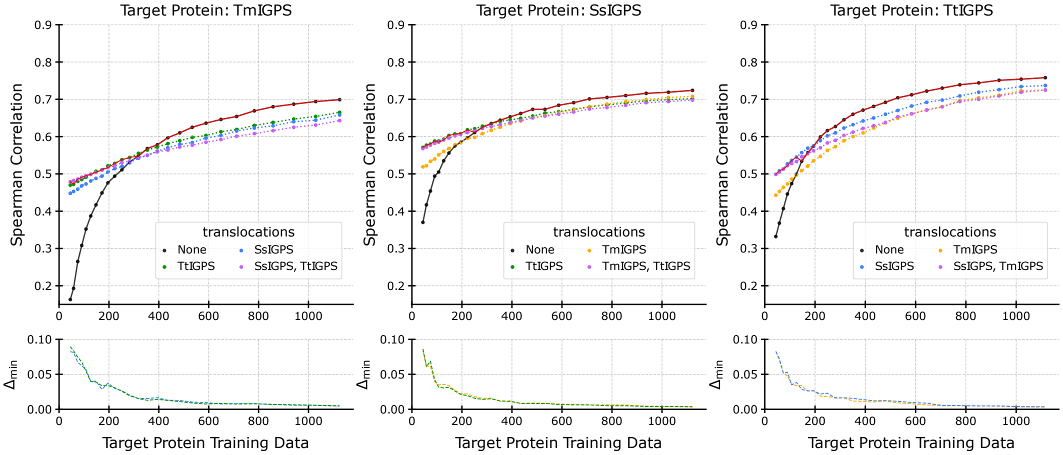

Figure S16.3: IGPS, ESM-1v pLM, and RF predictor.

Supplemental S17: GFP, ESM-1v, SVR - Lasso - RF, Statistical-Greedy

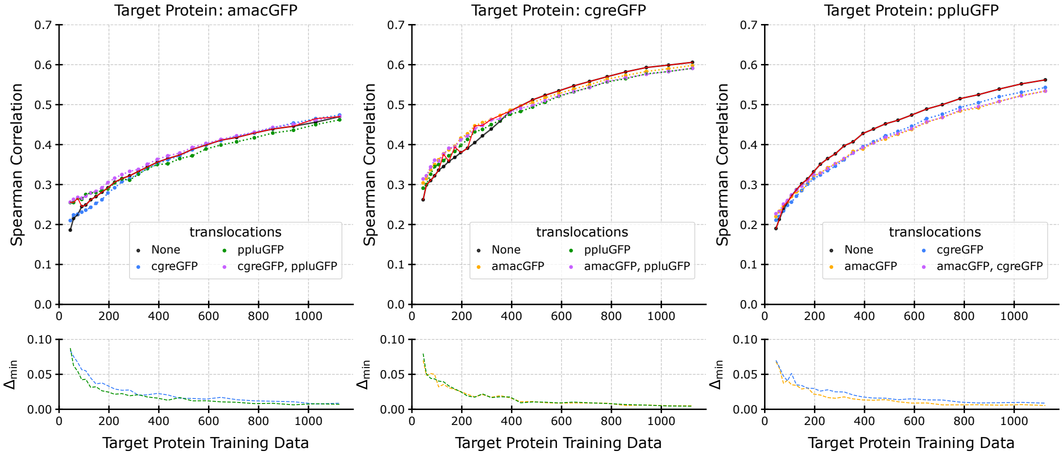

Figure S17.1: GFP, ESM-1v pLM, and SVR predictor.

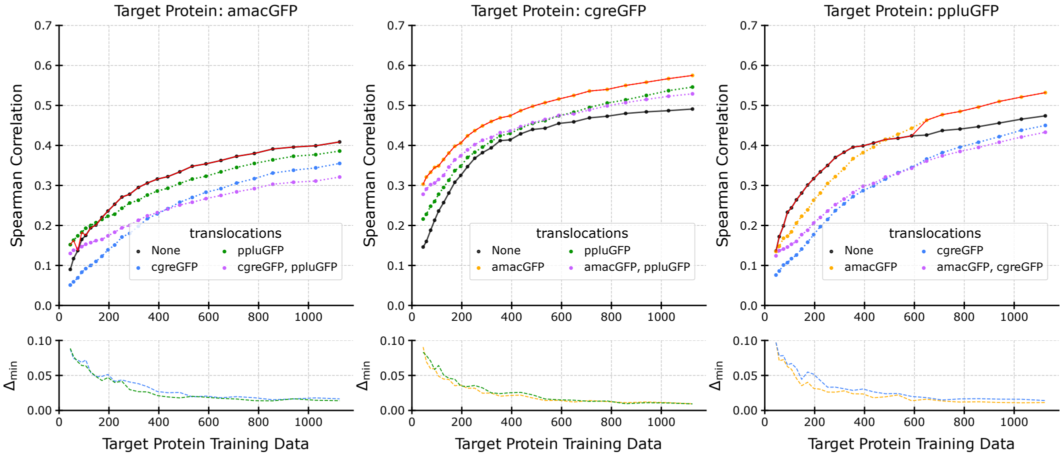

Figure S17.2: GFP, ESM-1v pLM, and Lasso predictor.

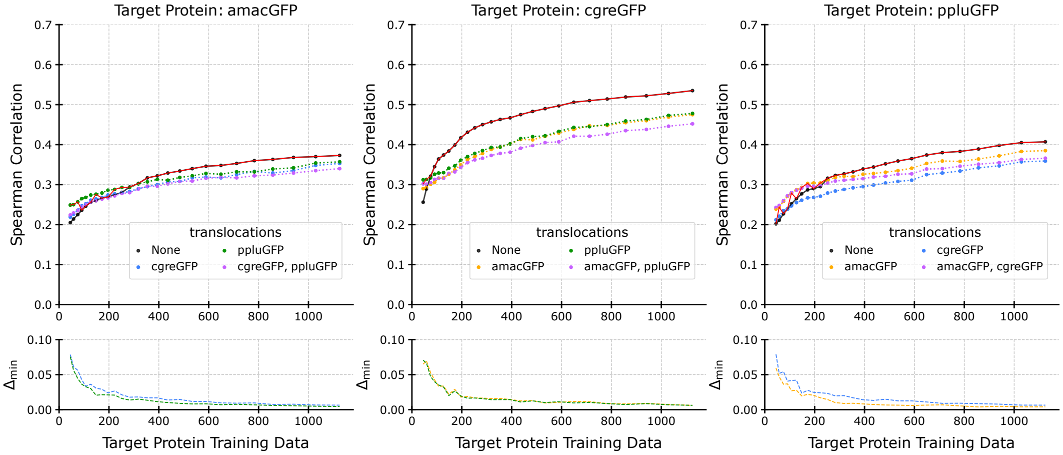

Figure S17.3: GFP, ESM-1v pLM, and RF predictor.

Supplemental S18: SARS-CoV-2 Spike protein Cell Entry, ESM2, SVR - Lasso - RF, Statistical-Greedy

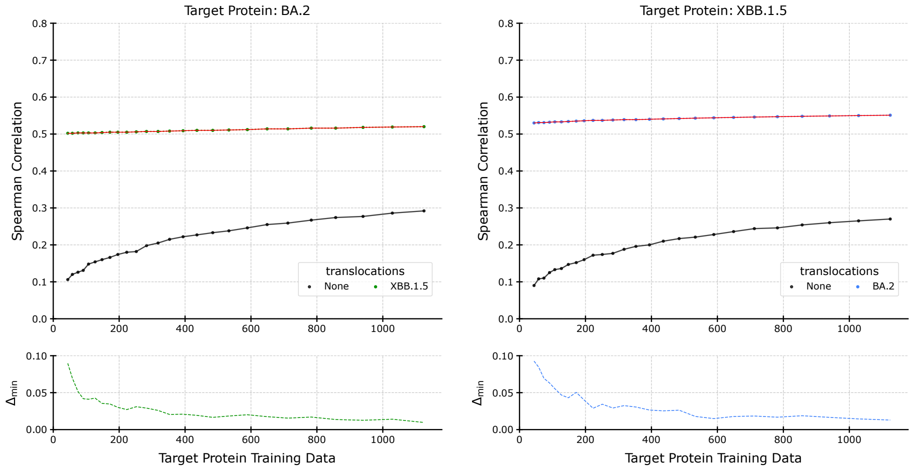

Figure S18.1: SARS-CoV-2 spike protein, ESM2 pLM, and SVR predictor.

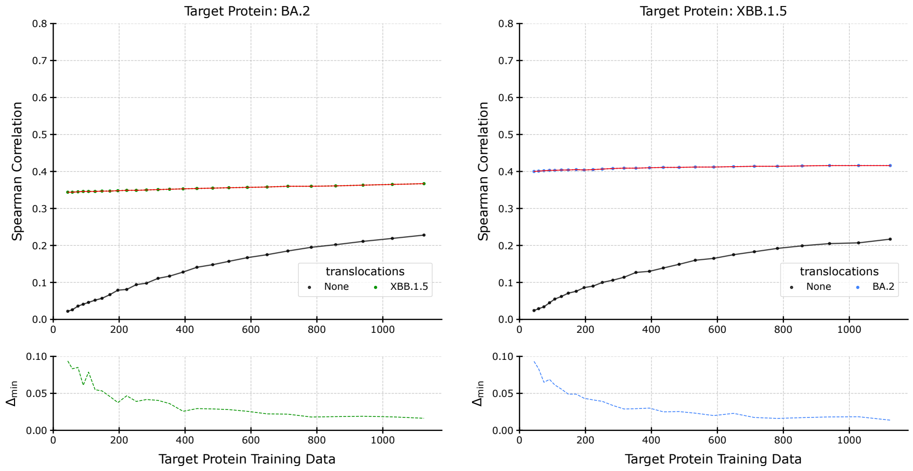

Figure S18.2: SARS-CoV-2 spike protein, ESM2 pLM, and Lasso predictor.

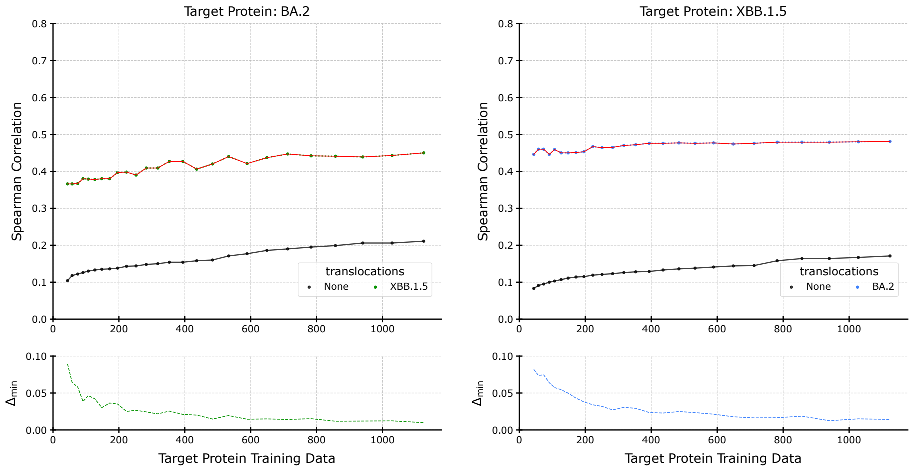

Figure S18.3: SARS-CoV-2 spike protein, ESM2 pLM, and RF predictor.

Supplemental S19: SARS-CoV-2 Spike protein ACE2 Binding, ESM2, SVR - Lasso - RF, Statistical-Greedy

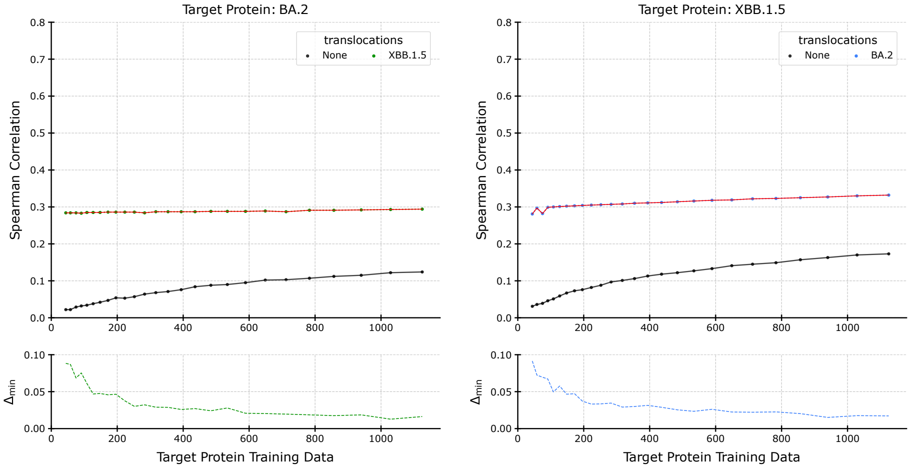

Figure S19.1: SARS-CoV-2 spike protein ACE2 Binding, ESM2 pLM, and SVR predictor.

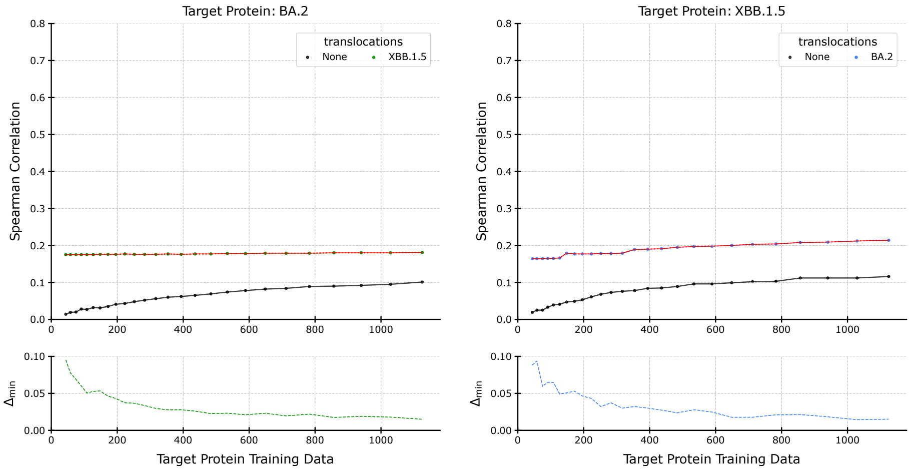

Figure S19.2: SARS-CoV-2 spike protein ACE2 Binding, ESM2 pLM, and Lasso predictor.

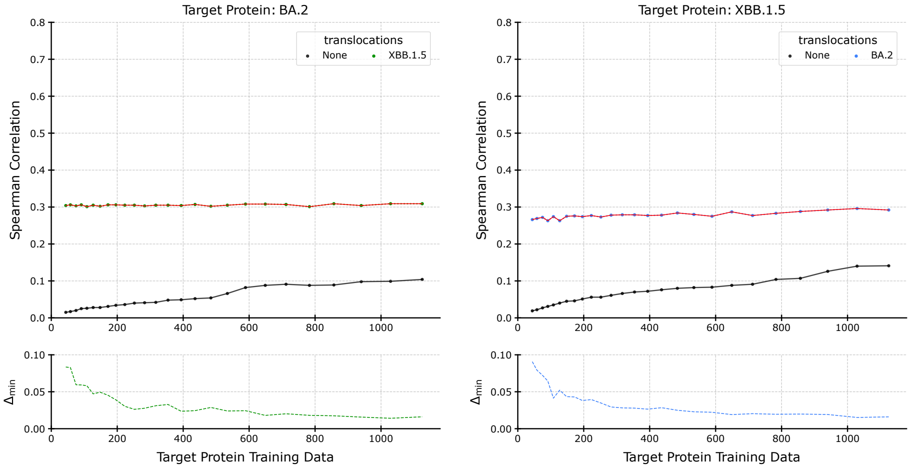

Figure S19.3: SARS-CoV-2 spike protein ACE2 Binding, ESM2 pLM, and RF predictor.

Supplemental S20: IGPS, ESM2, SVR - Lasso - RF, Statistical-Greedy

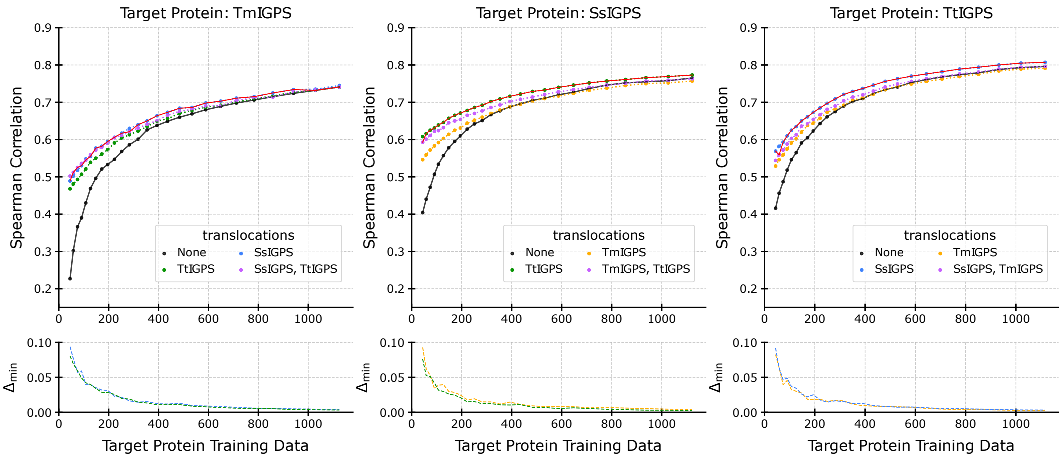

Figure S20.1: IGPS, ESM2 pLM, and SVR predictor.

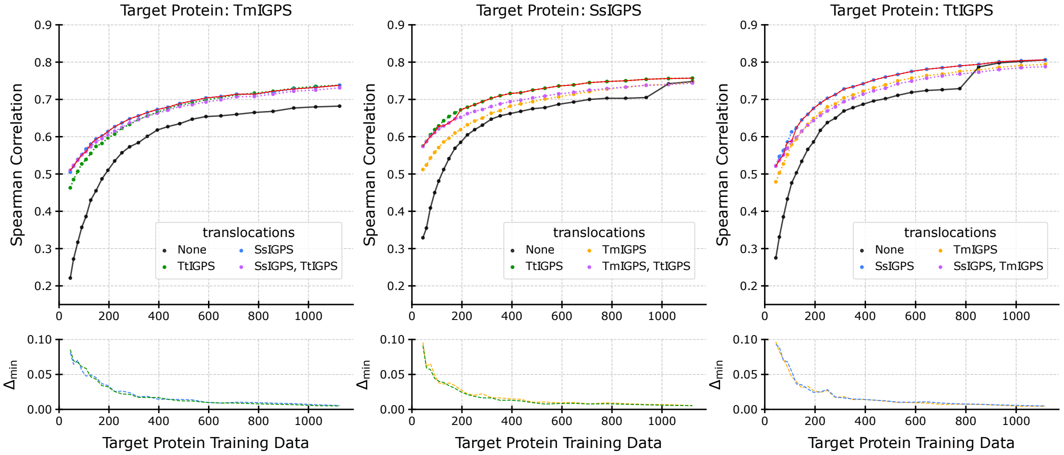

Figure S20.2: IGPS, ESM2 pLM, and Lasso predictor.

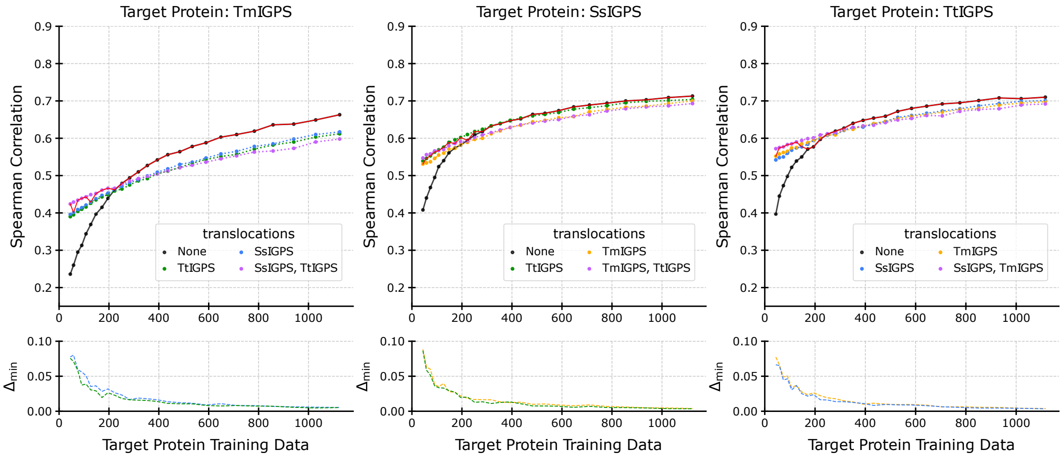

Figure S20.3: IGPS, ESM2 pLM, and RF predictor.

Supplemental S21: GFP, ESM2, SVR - Lasso - RF, Statistical-Greedy

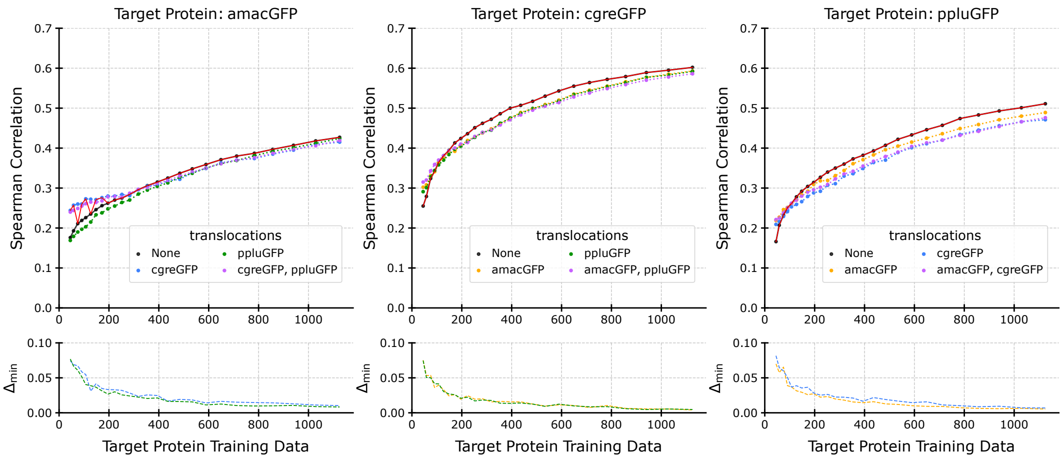

Figure S21.1: GFP, ESM2 pLM, and SVR predictor.

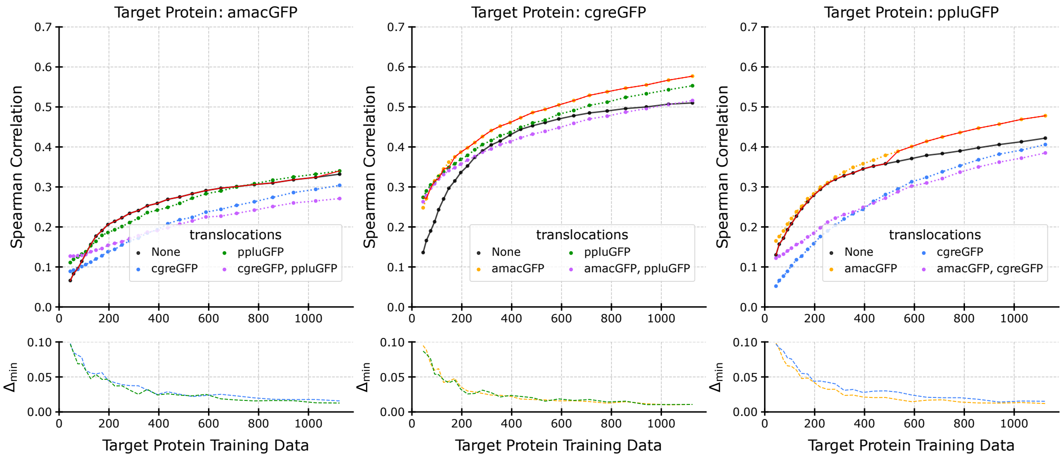

Figure S21.2: GFP, ESM2 pLM, and Lasso predictor.

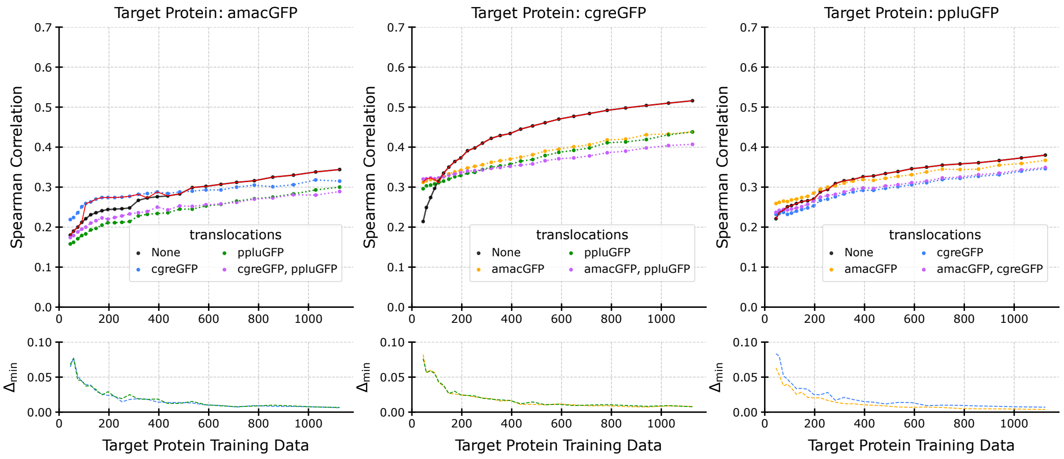

Figure S21.3: GFP, ESM2 pLM, and RF predictor.

Supplemental S22: SARS-CoV-2 Spike protein Cell Entry, ESM-1v, SVR, Statistical-Greedy - Individual-Greedy - Individual-Select

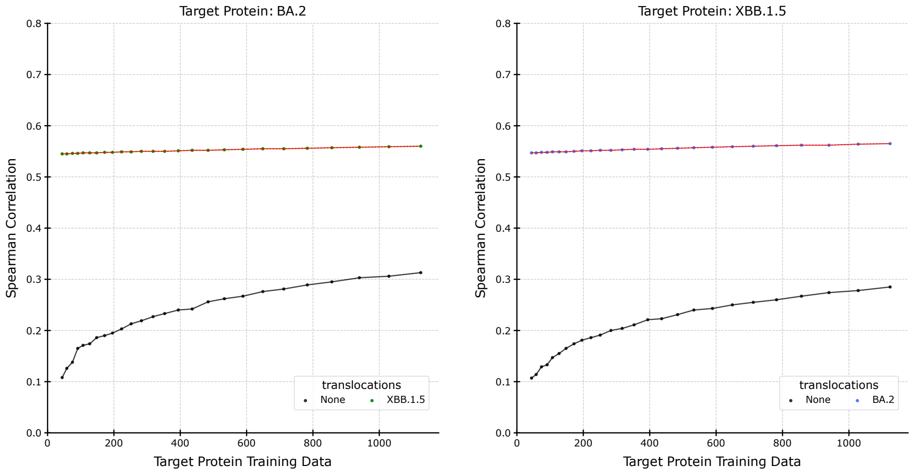

Figure S22.1: SARS-CoV-2 Spike protein Cell Entry, ESM-1v pLM, and SVR predictor.

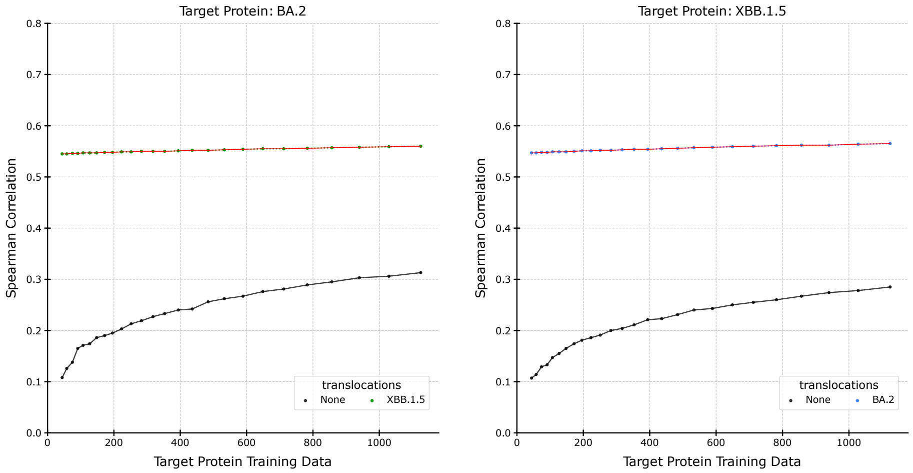

Figure S22.2: SARS-CoV-2 Spike protein Cell Entry, ESM-1v pLM, and SVR predictor.

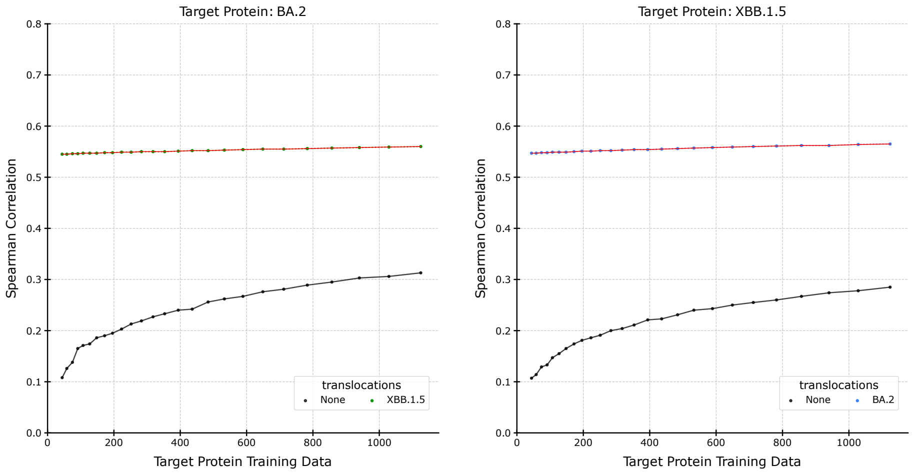

Figure S22.3: SARS-CoV-2 Spike protein Cell Entry, ESM-1v pLM, and SVR predictor.

Supplemental S23: SARS-CoV-2 Spike protein Cell Entry, ESM-1v, Lasso, Statistical-Greedy - Individual-Greedy - Individual-Select

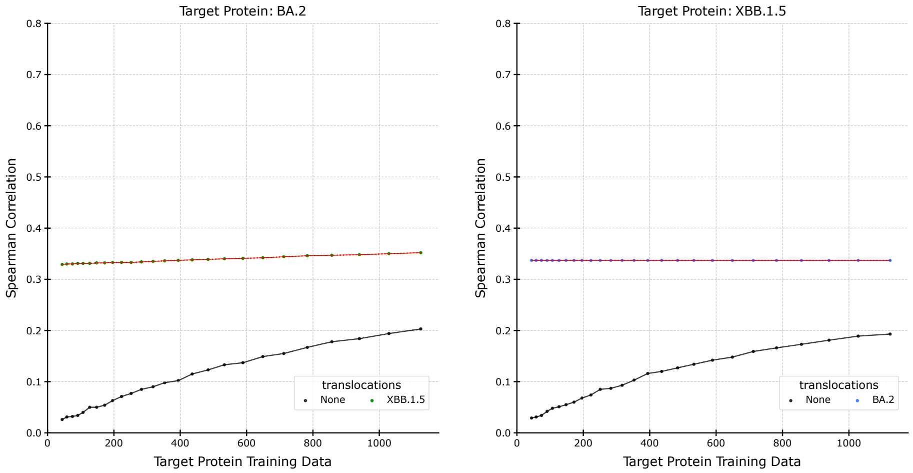

Figure S23.1: SARS-CoV-2 Spike protein Cell Entry, ESM-1v pLM, and Lasso predictor.

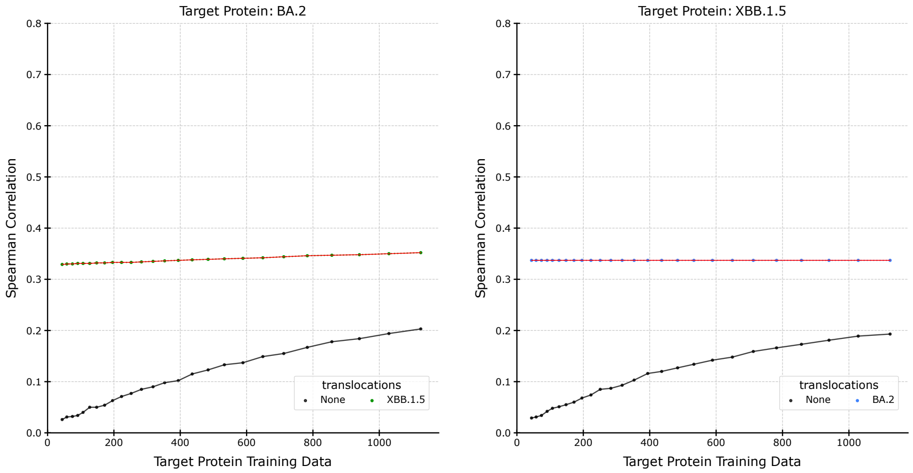

Figure S23.2: SARS-CoV-2 Spike protein Cell Entry, ESM-1v pLM, and Lasso predictor.

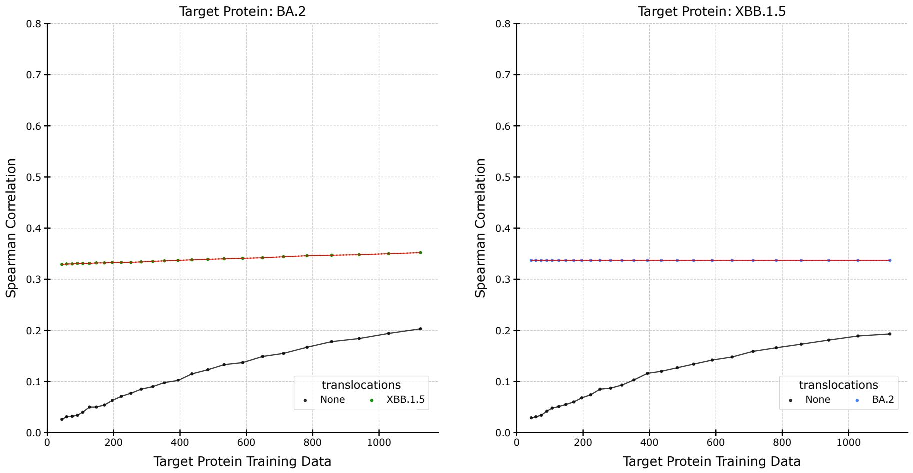

Figure S23.3: SARS-CoV-2 Spike protein Cell Entry, ESM-1v pLM, and Lasso predictor.

Supplemental S24: SARS-CoV-2 Spike protein Cell Entry, ESM-1v, RF, Statistical-Greedy - Individual-Greedy - Individual-Select

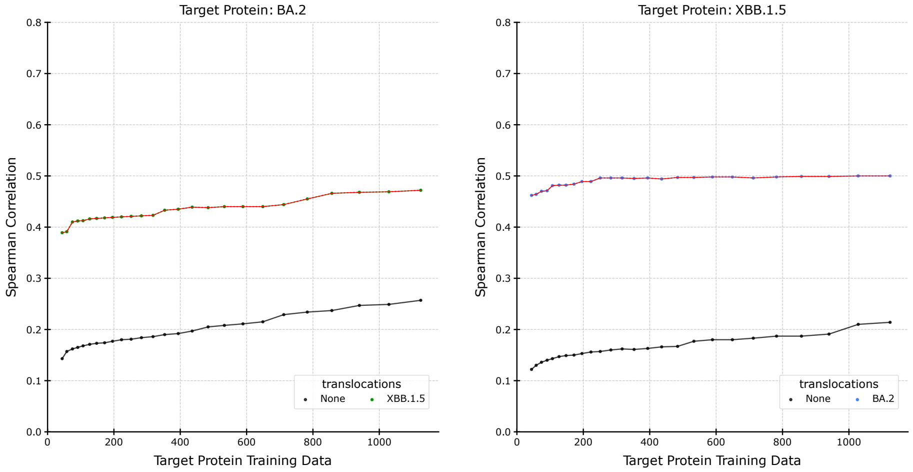

Figure S24.1: SARS-CoV-2 Spike protein Cell Entry, ESM-1v pLM, and RF predictor.

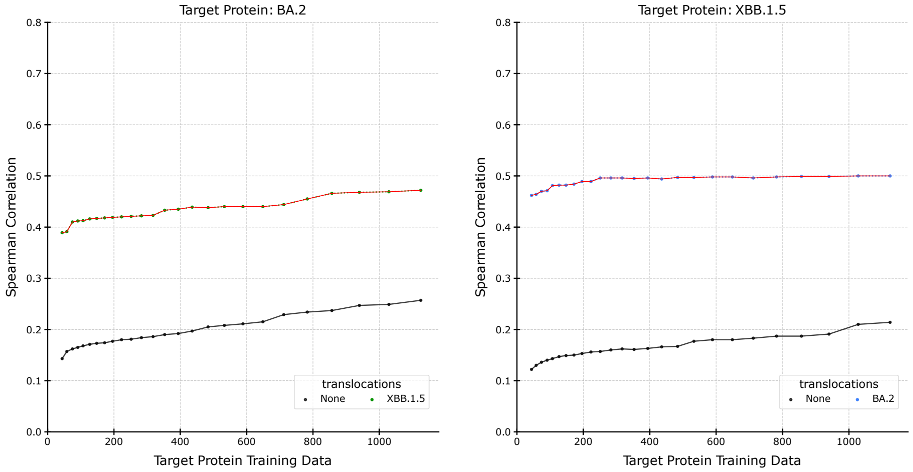

Figure S24.2: SARS-CoV-2 Spike protein Cell Entry, ESM-1v pLM, and RF predictor.

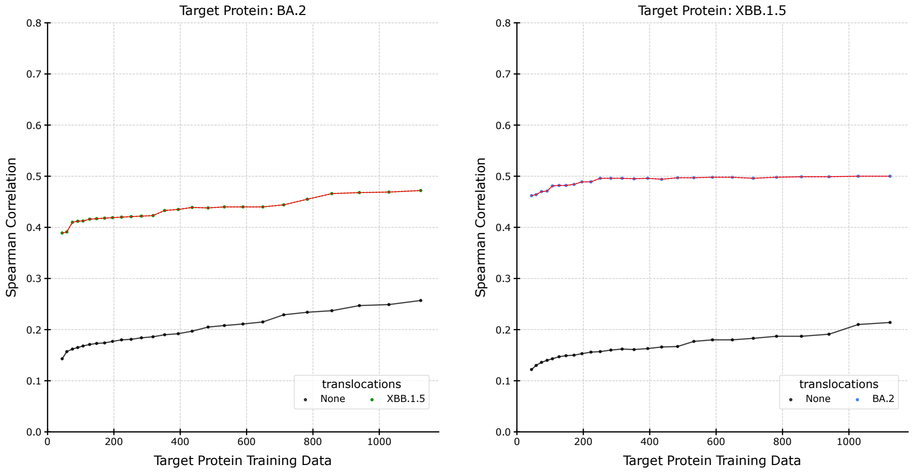

Figure S24.3: SARS-CoV-2 Spike protein Cell Entry, ESM-1v pLM, and RF predictor.

Supplemental S25: SARS-CoV-2 Spike protein ACE2 Binding, ESM-1v, SVR, Statistical-Greedy - Individual-Greedy - Individual-Select

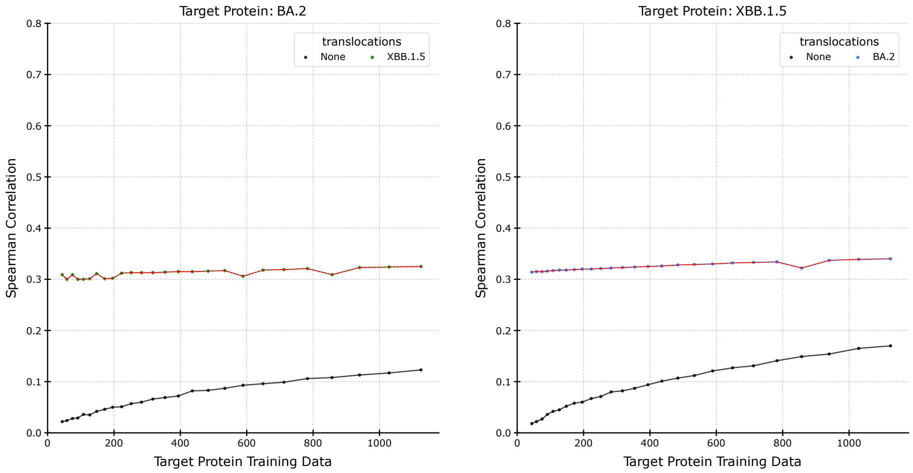

Figure S25.1: SARS-CoV-2 Spike protein ACE2 Binding, ESM-1v pLM, and SVR predictor.

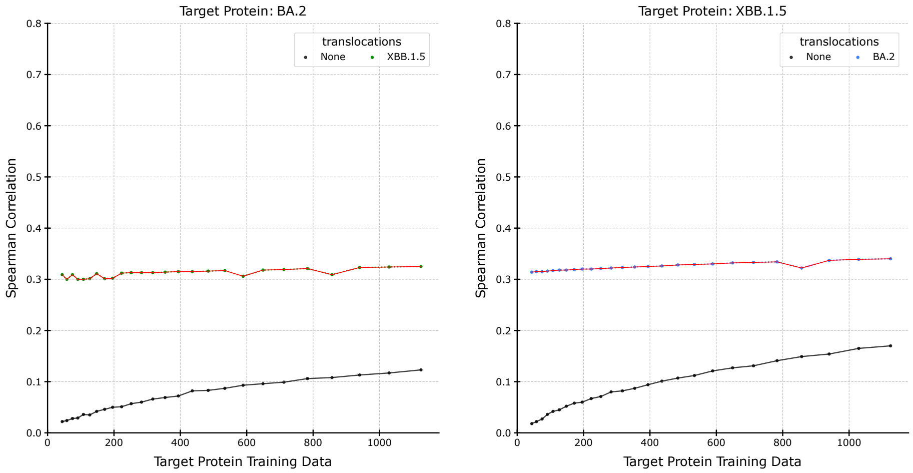

Figure S25.2: SARS-CoV-2 Spike protein ACE2 Binding, ESM-1v pLM, and SVR predictor.

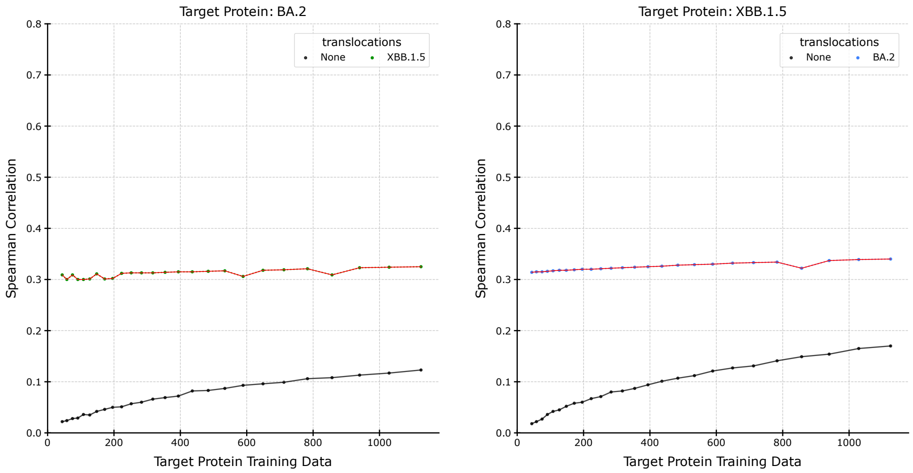

Figure S25.3: SARS-CoV-2 Spike protein ACE2 Binding, ESM-1v pLM, and SVR predictor.

Supplemental S26: SARS-CoV-2 Spike protein ACE2 Binding, ESM-1v, Lasso, Statistical-Greedy - Individual-Greedy - Individual-Select

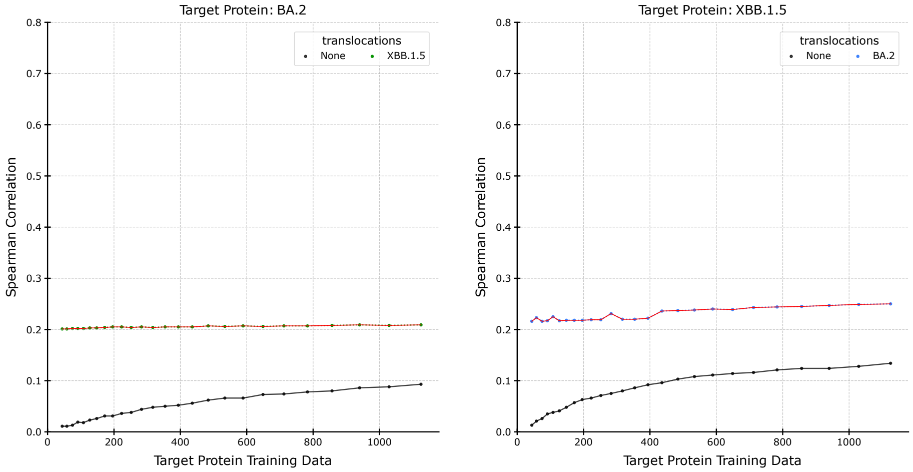

Figure S26.1: SARS-CoV-2 Spike protein ACE2 Binding, ESM-1v pLM, and Lasso predictor.

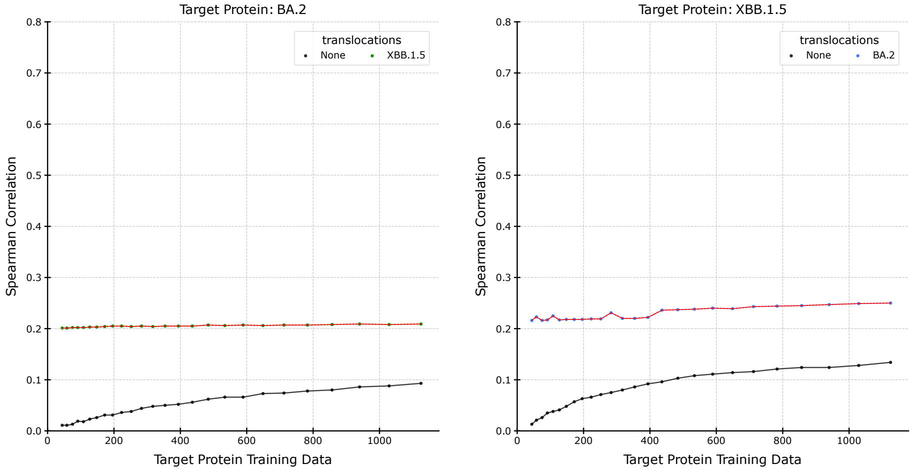

Figure S26.2: SARS-CoV-2 Spike protein ACE2 Binding, ESM-1v pLM, and Lasso predictor.

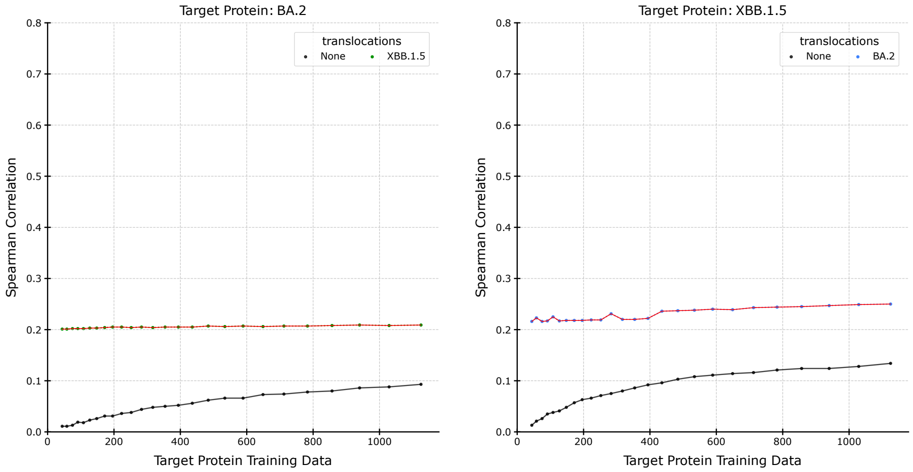

Figure S26.3: SARS-CoV-2 Spike protein ACE2 Binding, ESM-1v pLM, and Lasso predictor.

Supplemental S27: SARS-CoV-2 Spike protein ACE2 Binding, ESM-1v, RF, Statistical-Greedy - Individual-Greedy - Individual-Select

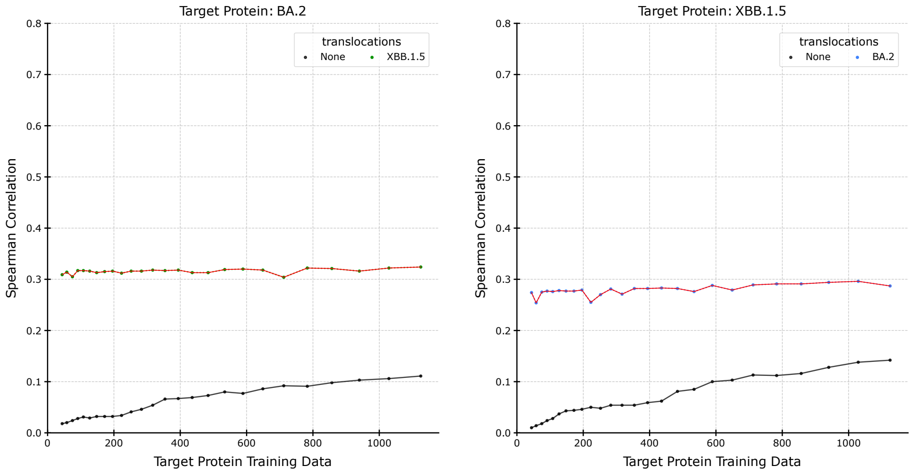

Figure S27.1: SARS-CoV-2 Spike protein ACE2 Binding, ESM-1v pLM, and RF predictor.

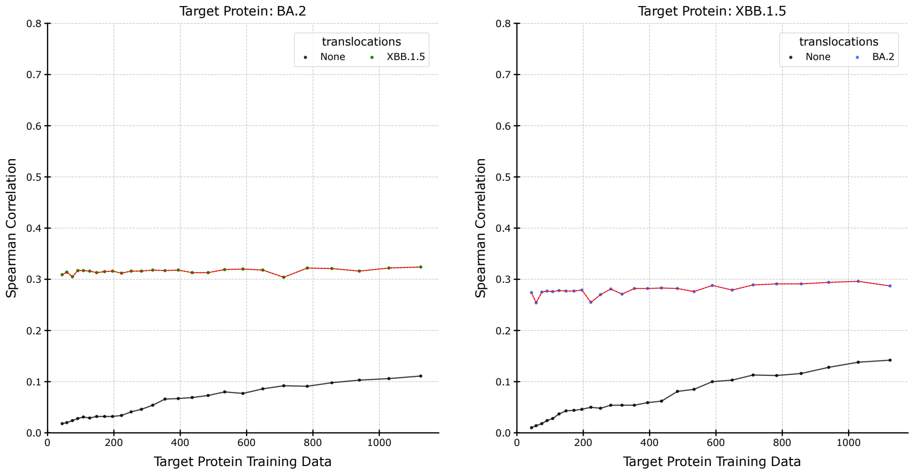

Figure S27.2: SARS-CoV-2 Spike protein ACE2 Binding, ESM-1v pLM, and RF predictor.

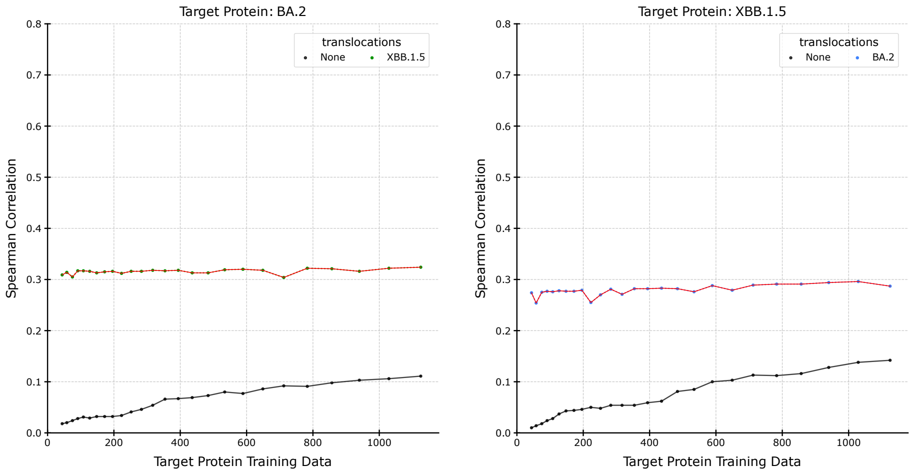

Figure S27.3: SARS-CoV-2 Spike protein ACE2 Binding, ESM-1v pLM, and RF predictor.

Supplemental S28: IGPS, ESM-1v, SVR, Statistical-Greedy - Individual-Greedy - Individual-Select

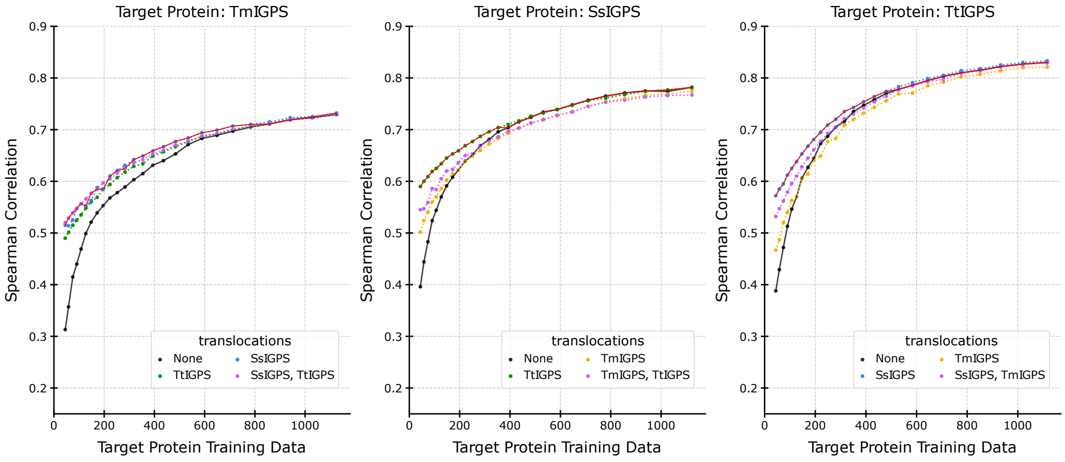

Figure S28.1: IGPS, ESM-1v pLM, and SVR predictor.

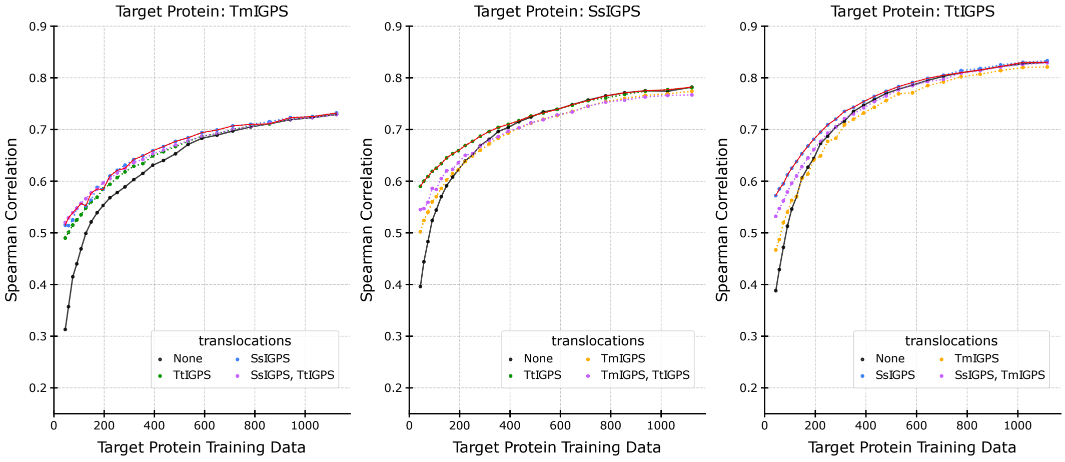

Figure S28.2: IGPS, ESM-1v pLM, and SVR predictor.

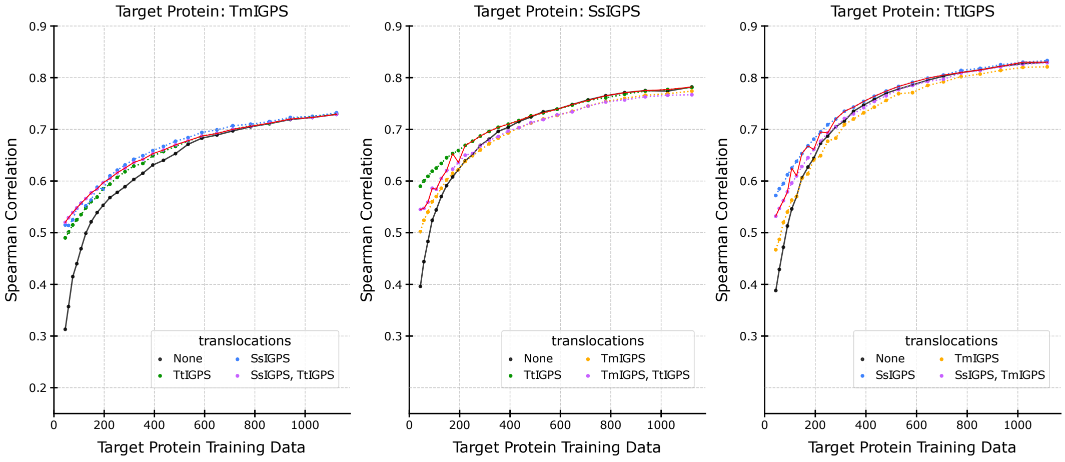

Figure S28.3: IGPS, ESM-1v pLM, and SVR predictor.

Supplemental S29: IGPS, ESM-1v, Lasso, Statistical-Greedy - Individual-Greedy - Individual-Select

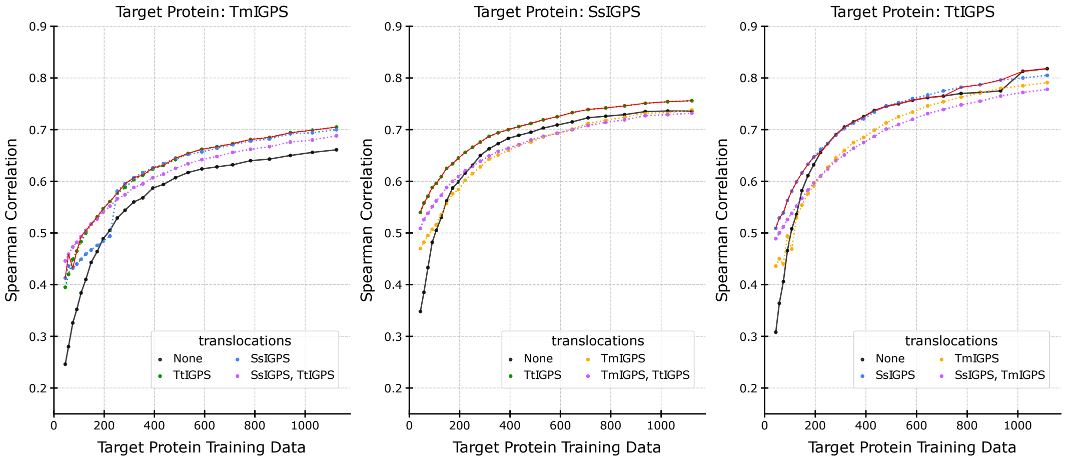

Figure S29.1: IGPS, ESM-1v pLM, and Lasso predictor.

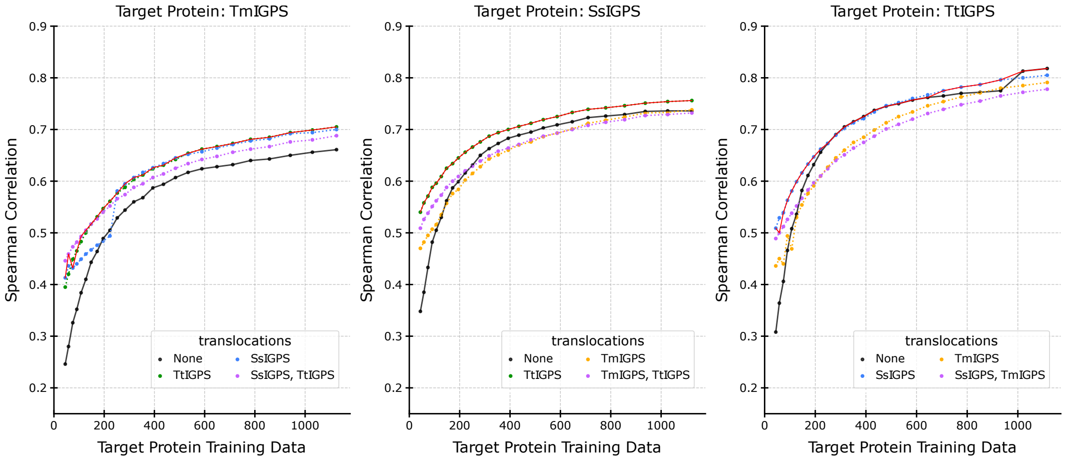

Figure S29.2: IGPS, ESM-1v pLM, and Lasso predictor.

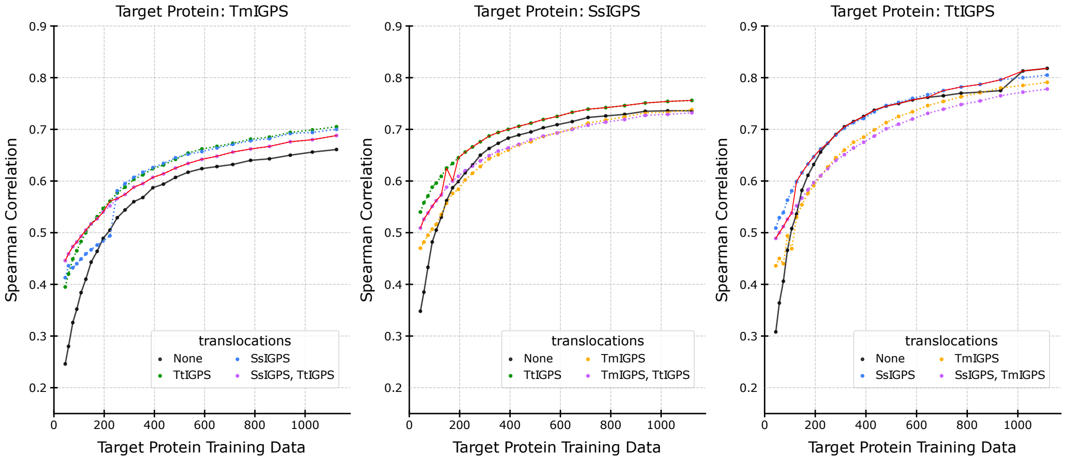

Figure S29.3: IGPS, ESM-1v pLM, and Lasso predictor.

Supplemental S30: IGPS, ESM-1v, RF, Statistical-Greedy - Individual-Greedy - Individual-Select

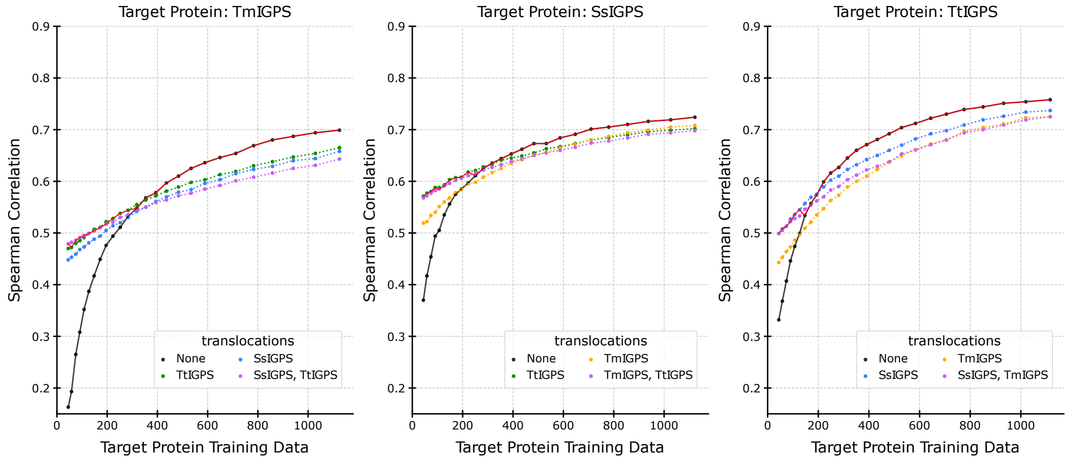

Figure S30.1: IGPS, ESM-1v pLM, and RF predictor.

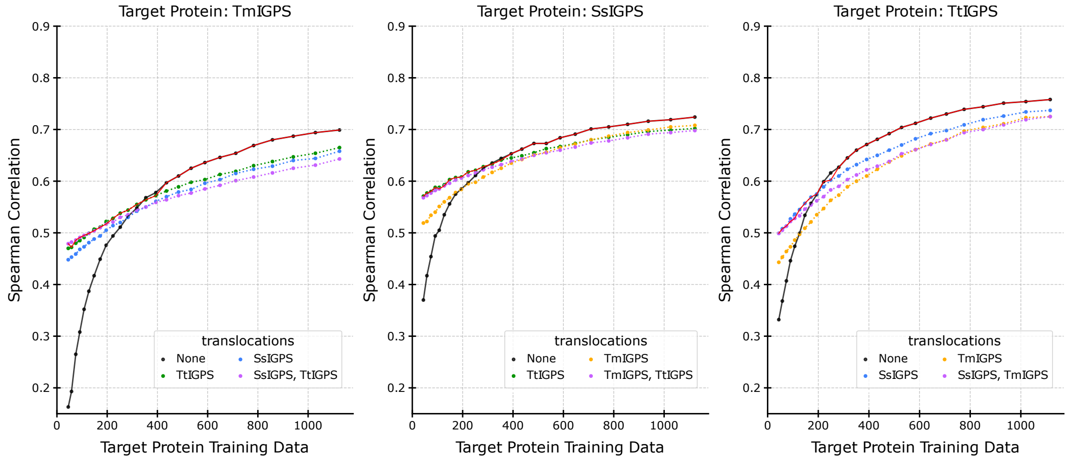

Figure S30.2: IGPS, ESM-1v pLM, and RF predictor.

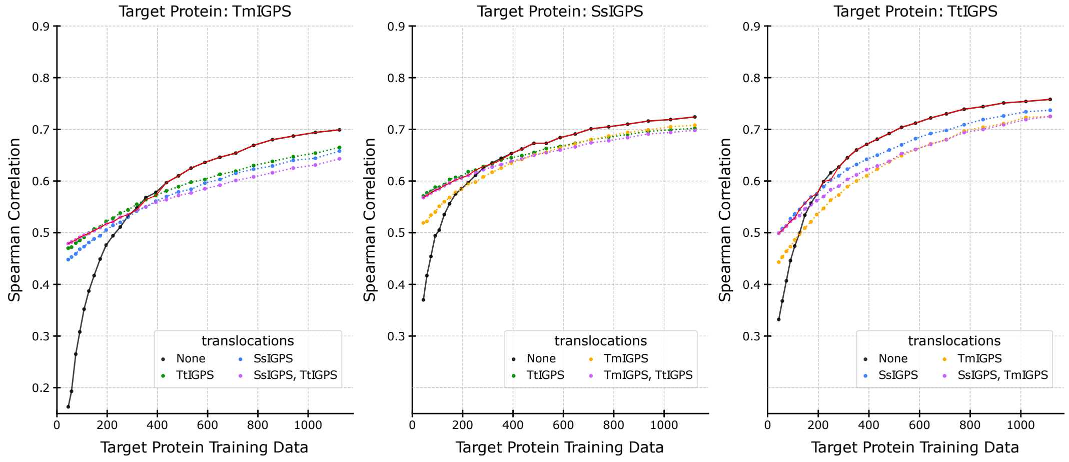

Figure S30.3: IGPS, ESM-1v pLM, and RF predictor.

Supplemental S31: GFP, ESM-1v, SVR, Statistical-Greedy - Individual-Greedy - Individual-Select

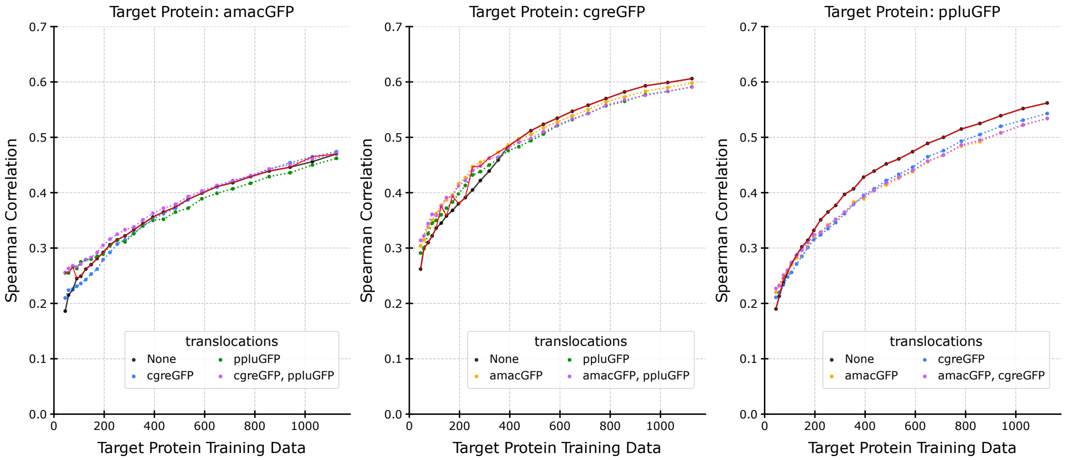

Figure S31.1: GFP, ESM-1v pLM, and SVR predictor.

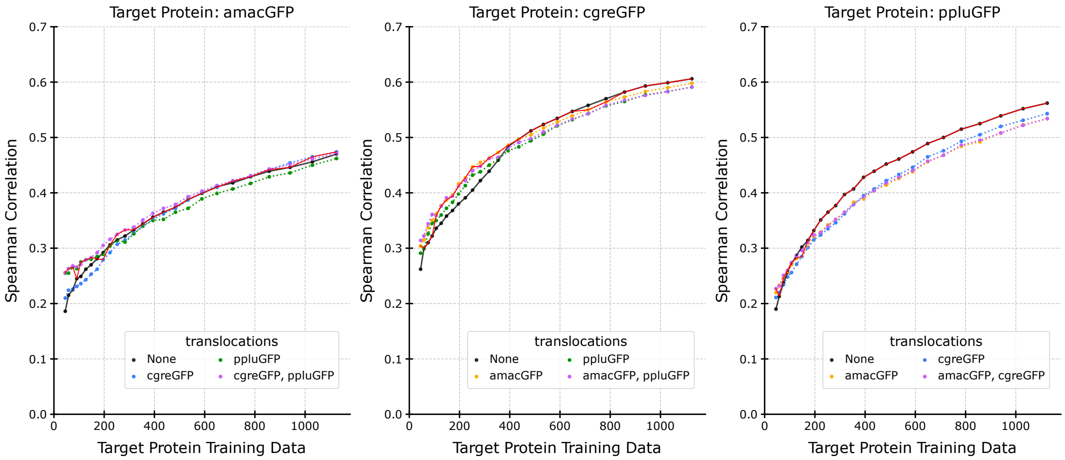

Figure S31.2: GFP, ESM-1v pLM, and SVR predictor.

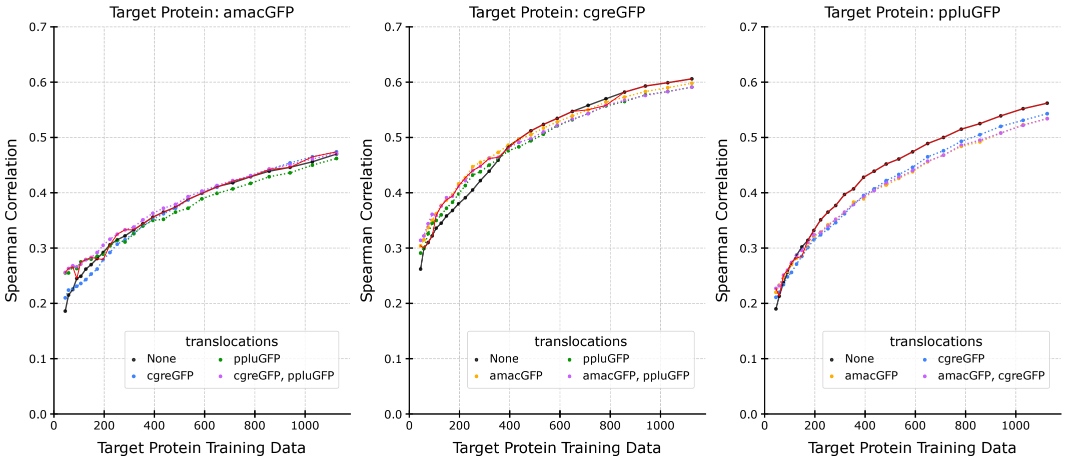

Figure S31.3: GFP, ESM-1v pLM, and SVR predictor.

Supplemental S32: GFP, ESM-1v, Lasso, Statistical-Greedy - Individual-Greedy - Individual-Select

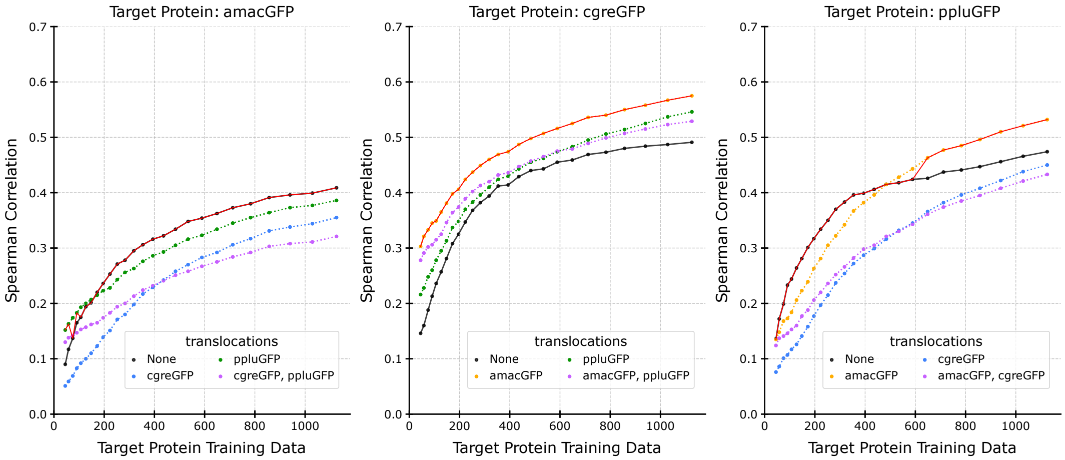

Figure S32.1: GFP, ESM-1v pLM, and Lasso predictor.

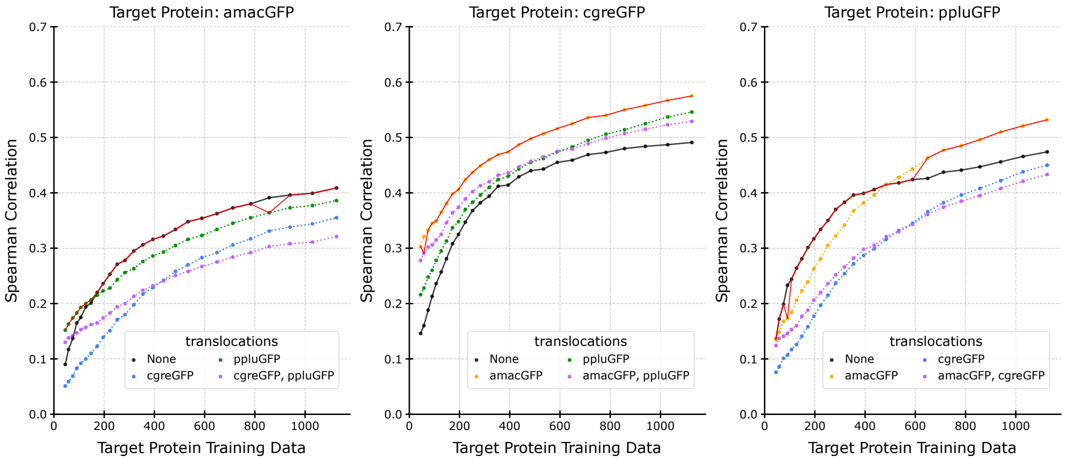

Figure S32.2: GFP, ESM-1v pLM, and Lasso predictor.

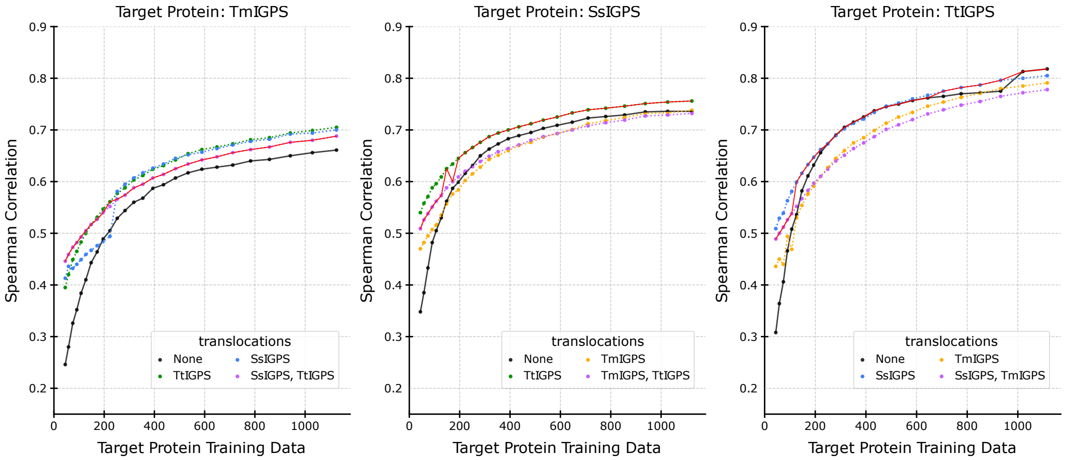

Figure S32.3: GFP, ESM-1v pLM, and Lasso predictor.

Supplemental S33: GFP, ESM-1v, RF, Statistical-Greedy - Individual-Greedy - Individual-Select

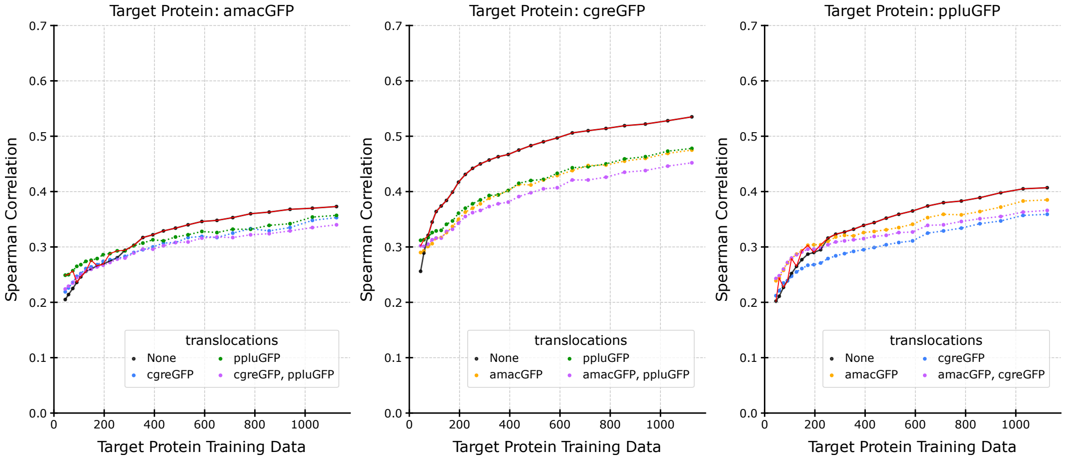

Figure S33.1: GFP, ESM-1v pLM, and RF predictor.

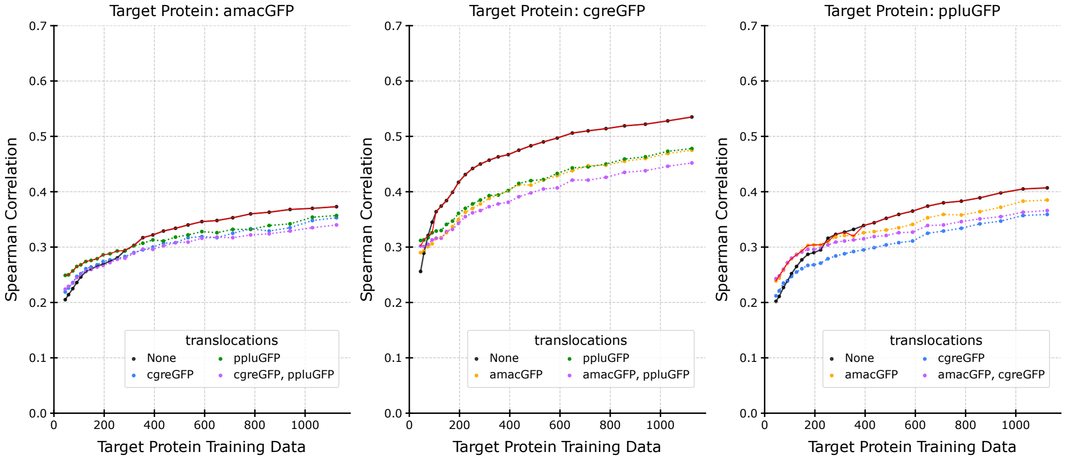

Figure S33.2: GFP, ESM-1v pLM, and RF predictor.

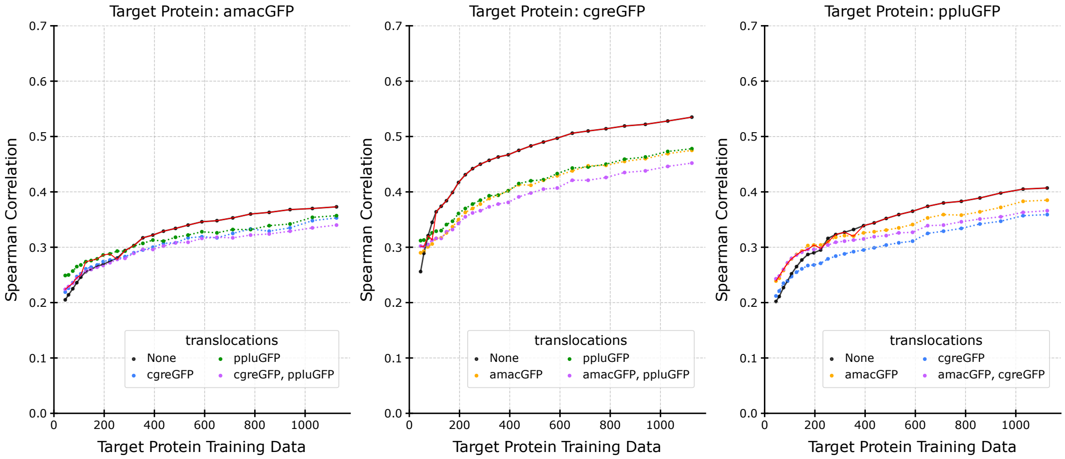

Figure S33.3: GFP, ESM-1v pLM, and RF predictor.

Supplemental S34: SARS-CoV-2 Spike protein Cell Entry, ESM2, SVR, Statistical-Greedy - Individual-Greedy - Individual-Select

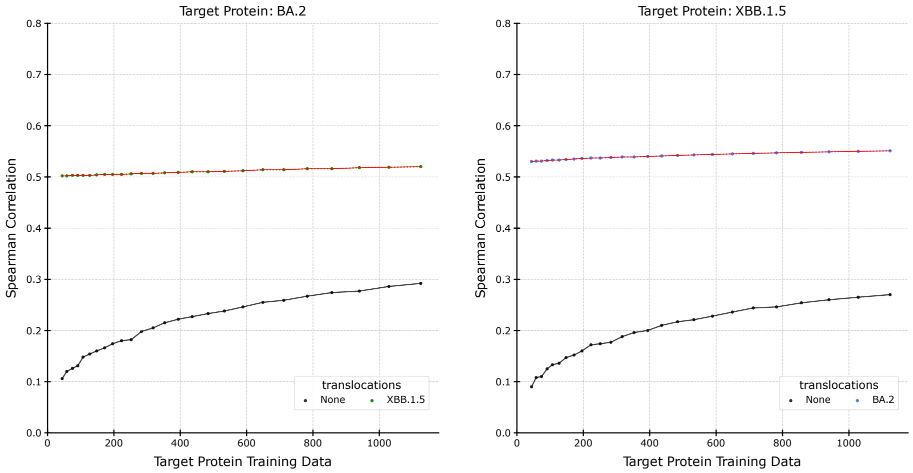

Figure S34.1: SARS-CoV-2 Spike protein Cell Entry, ESM2 pLM, and SVR predictor.

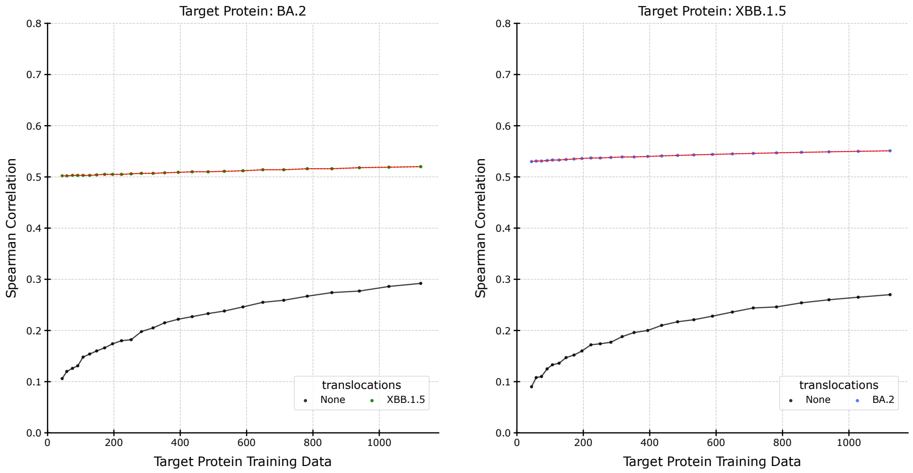

Figure S34.2: SARS-CoV-2 Spike protein Cell Entry, ESM2 pLM, and SVR predictor.

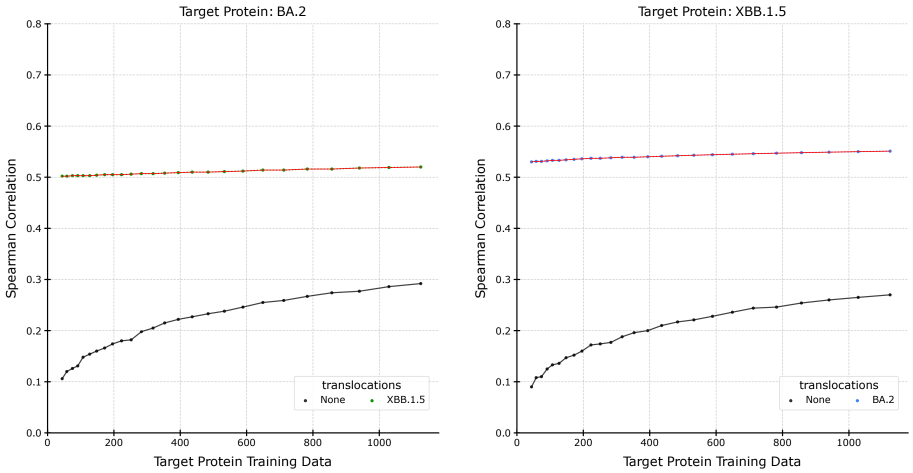

Figure S34.3: SARS-CoV-2 Spike protein Cell Entry, ESM2 pLM, and SVR predictor.

Supplemental S35: SARS-CoV-2 Spike protein Cell Entry, ESM2, Lasso, Statistical-Greedy - Individual-Greedy - Individual-Select

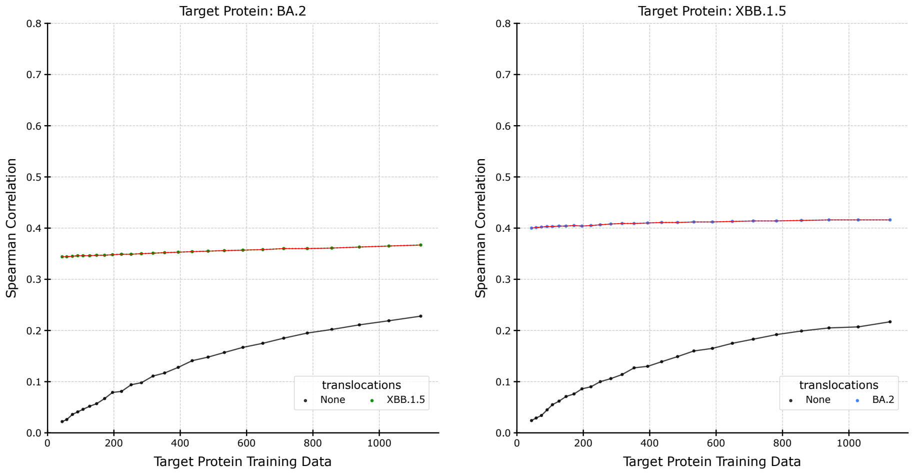

Figure S35.1: SARS-CoV-2 Spike protein Cell Entry, ESM2 pLM, and lasso predictor.

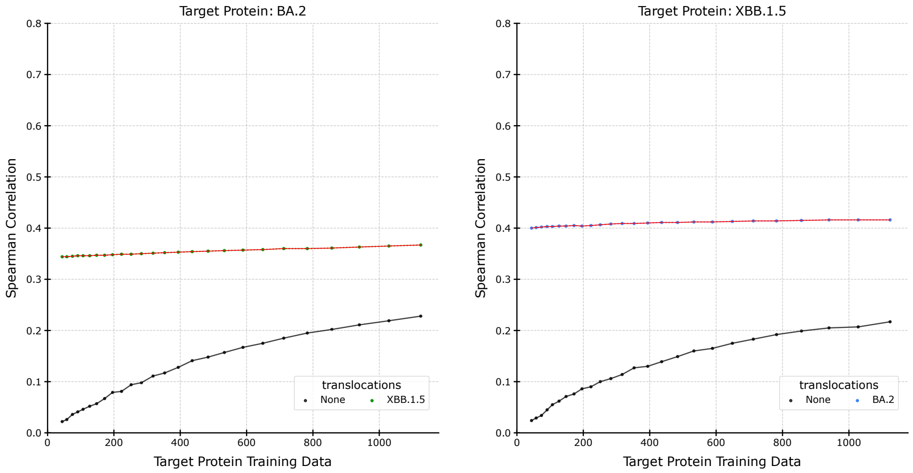

Figure S35.2: SARS-CoV-2 Spike protein Cell Entry, ESM2 pLM, and Lasso predictor.

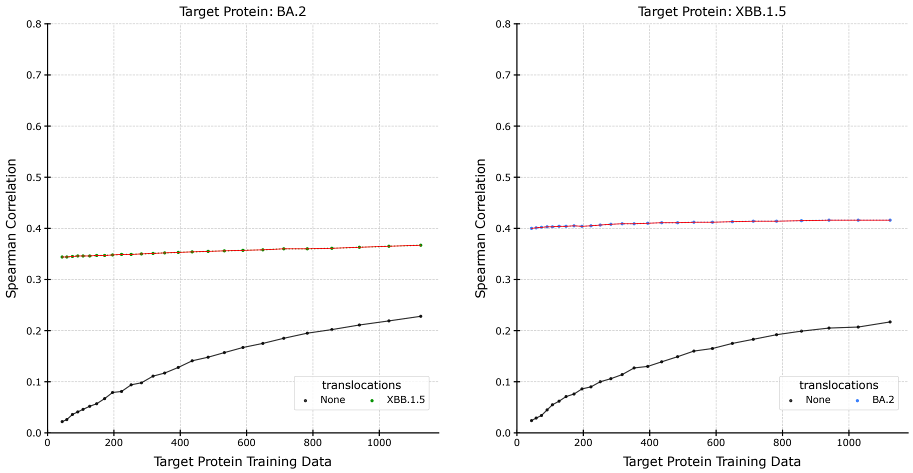

Figure S35.3: SARS-CoV-2 Spike protein Cell Entry, ESM2 pLM, and lasso predictor.

Supplemental S36: SARS-CoV-2 Spike protein Cell Entry, ESM2, RF, Statistical-Greedy - Individual-Greedy - Individual-Select

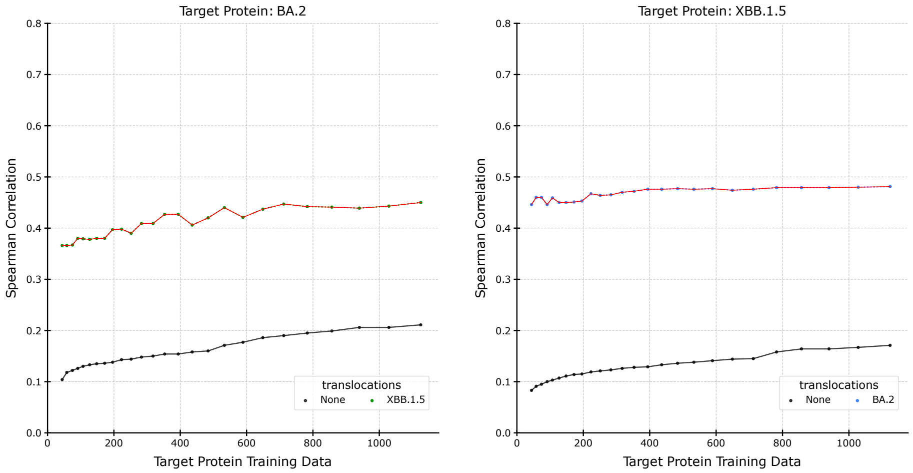

Figure S36.1: SARS-CoV-2 Spike protein Cell Entry, ESM2 pLM, and RF predictor.

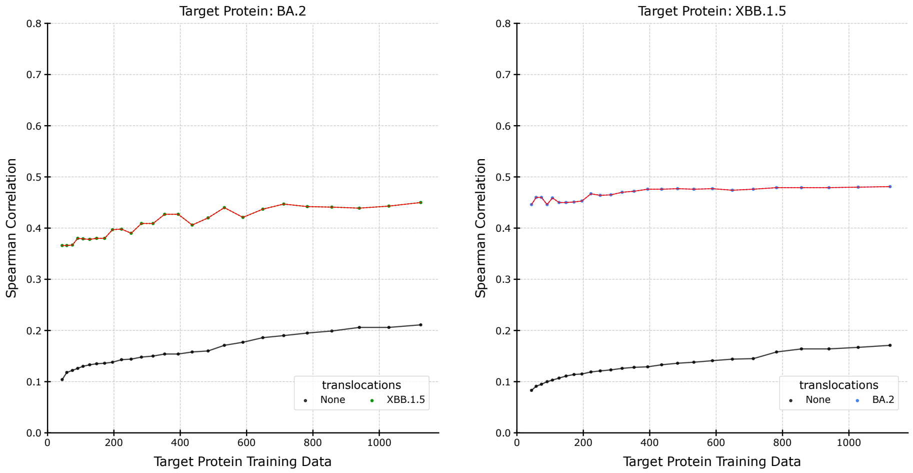

Figure S36.2: SARS-CoV-2 Spike protein Cell Entry, ESM2 pLM, and RF predictor.

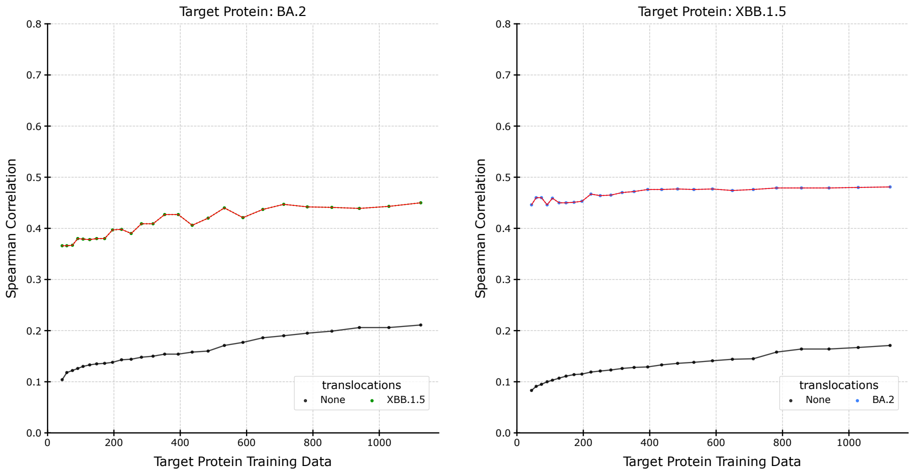

Figure S36.3: SARS-CoV-2 Spike protein Cell Entry, ESM2 pLM, and RF predictor.

Supplemental S37: SARS-CoV-2 Spike protein ACE2 Binding, ESM2, SVR, Statistical-Greedy - Individual-Greedy - Individual-Select

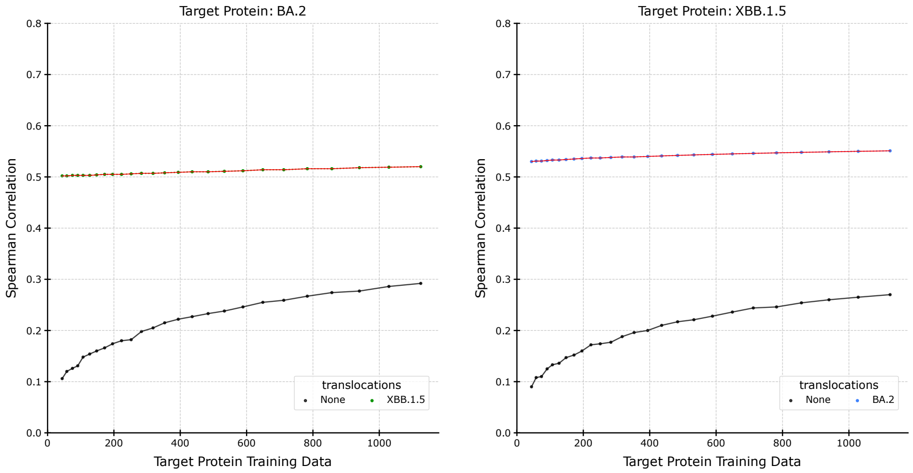

Figure S37.1: SARS-CoV-2 Spike protein ACE2 Binding, ESM2 pLM, and SVR predictor.

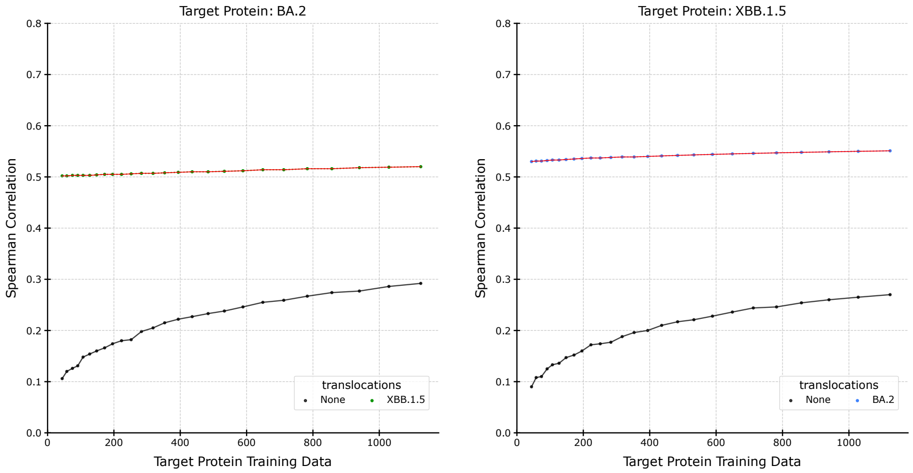

Figure S37.2: SARS-CoV-2 Spike protein ACE2 Binding, ESM2 pLM, and SVR predictor.

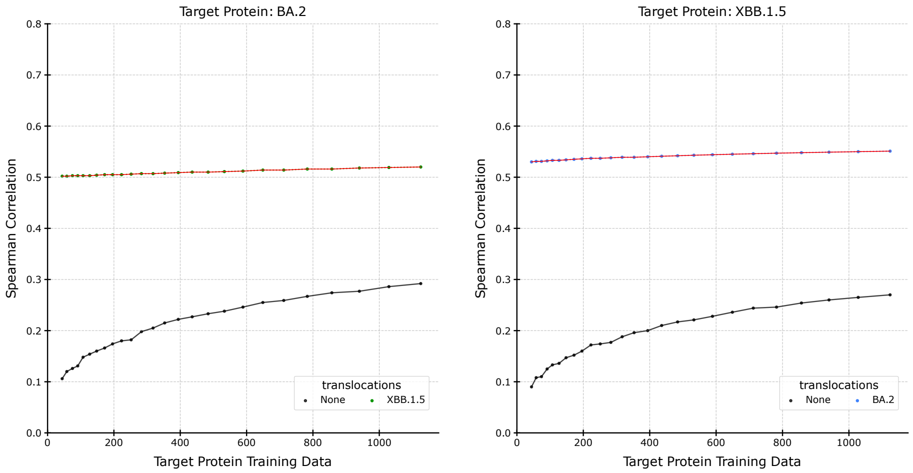

Figure S37.3: SARS-CoV-2 Spike protein ACE2 Binding, ESM2 pLM, and SVR predictor.

Supplemental S38: SARS-CoV-2 Spike protein ACE2 Binding, ESM2, Lasso, Statistical-Greedy - Individual-Greedy - Individual-Select

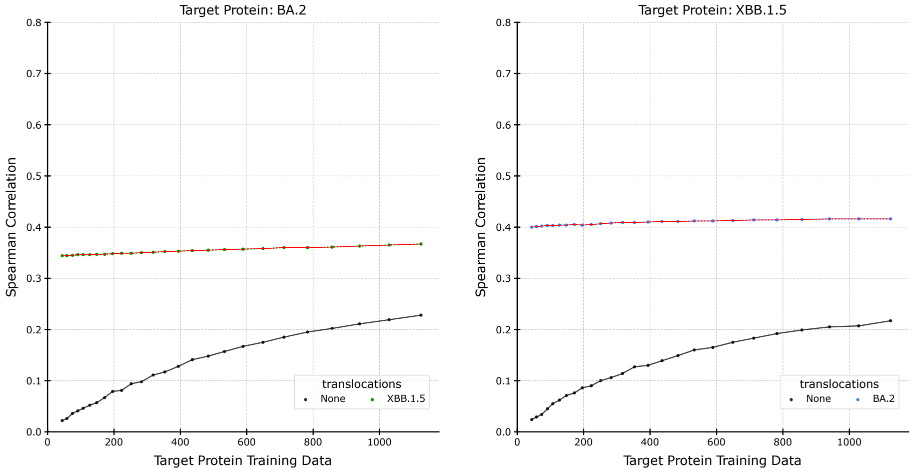

Figure S38.1: SARS-CoV-2 Spike protein ACE2 Binding, ESM2 pLM, and lasso predictor.

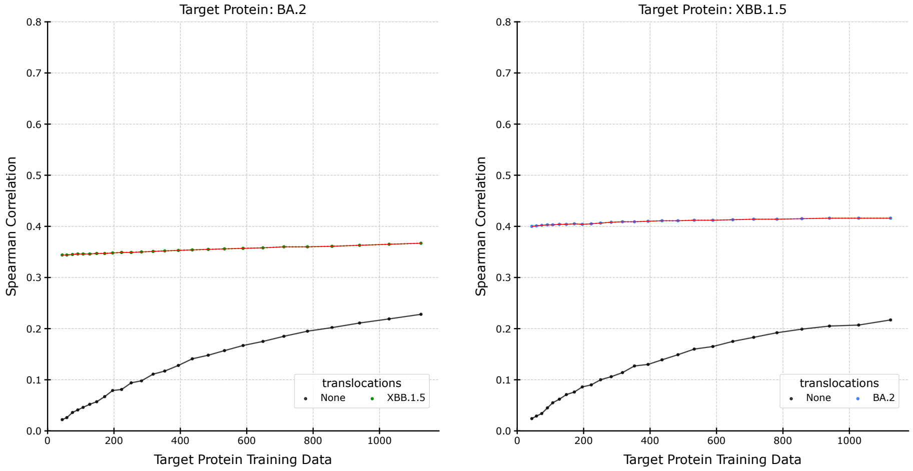

Figure S38.2: SARS-CoV-2 Spike protein ACE2 Binding, ESM2 pLM, and Lasso predictor.

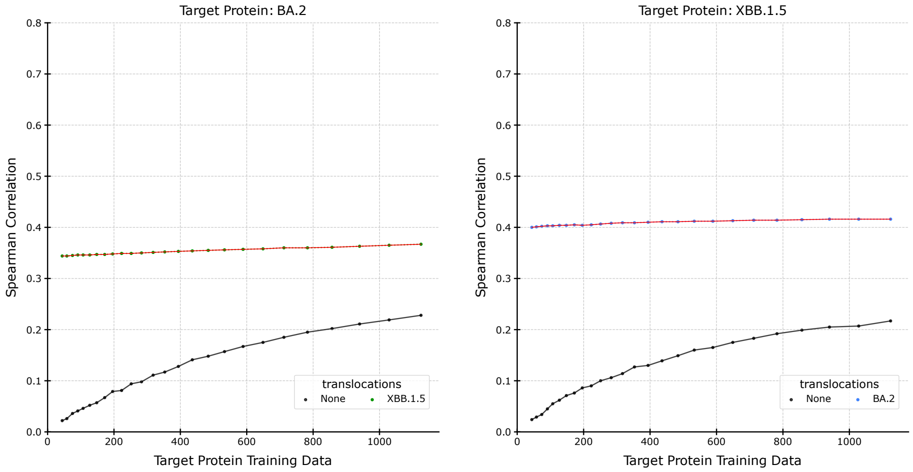

Figure S38.3: SARS-CoV-2 Spike protein ACE2 Binding, ESM2 pLM, and lasso predictor.

Supplemental S39: SARS-CoV-2 Spike protein ACE2 Binding, ESM2, RF, Statistical-Greedy - Individual-Greedy - Individual-Select

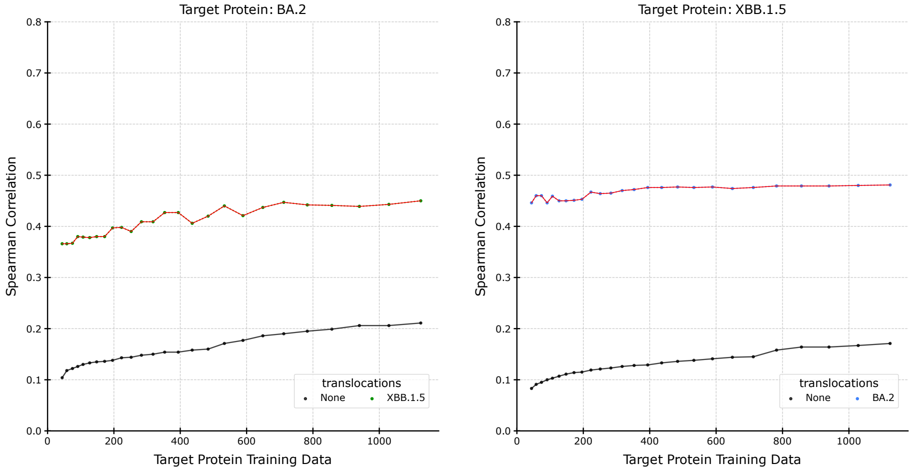

Figure S39.1: SARS-CoV-2 Spike protein ACE2 Binding, ESM2 pLM, and RF predictor.

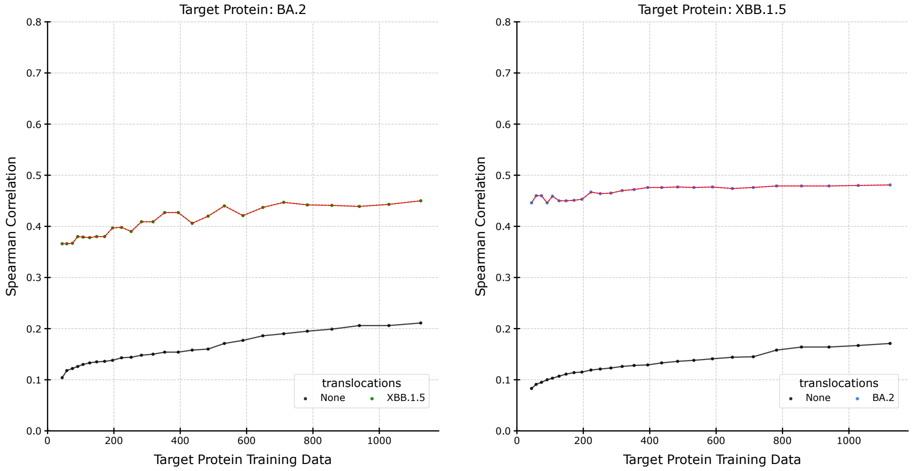

Figure S39.2: SARS-CoV-2 Spike protein ACE2 Binding, ESM2 pLM, and RF predictor.

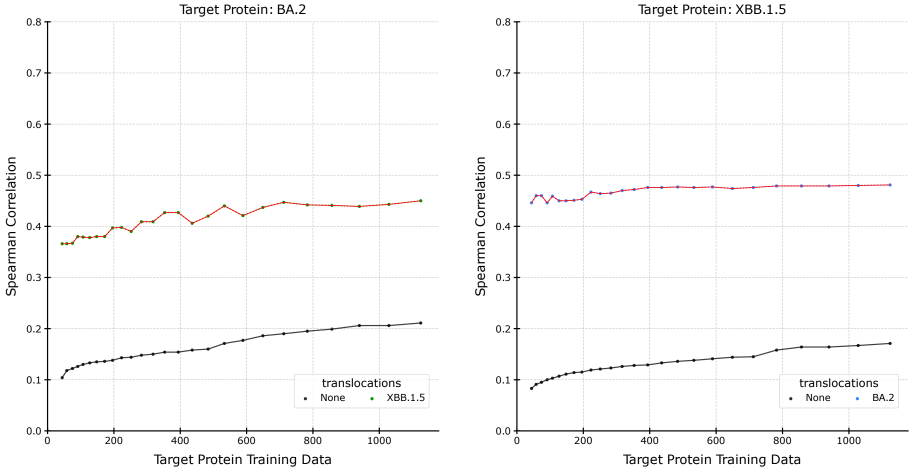

Figure S39.3: SARS-CoV-2 Spike protein ACE2 Binding, ESM2 pLM, and RF predictor.

Supplemental S40: IGPS, ESM2, SVR, Statistical-Greedy - Individual-Greedy - Individual-Select

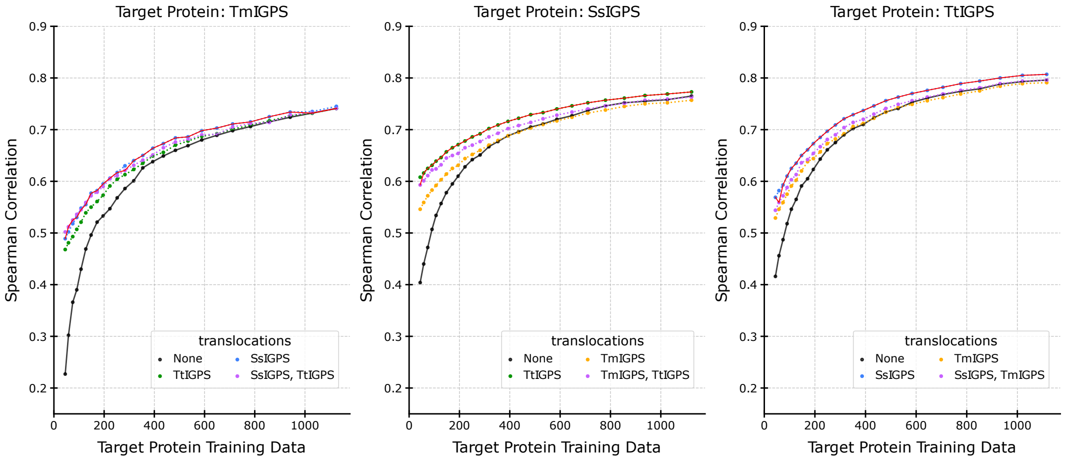

Figure S40.1: IGPS, ESM2 pLM, and SVR predictor.

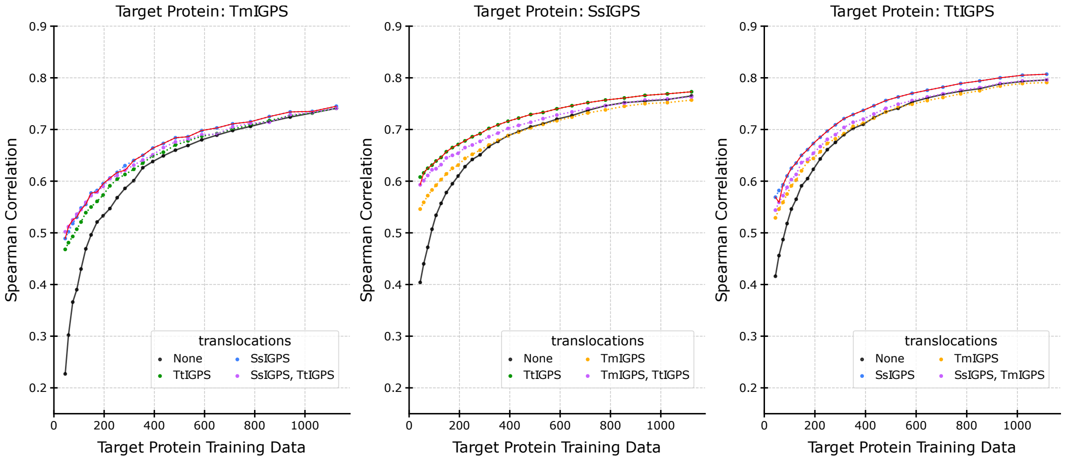

Figure S40.2: IGPS, ESM2 pLM, and SVR predictor.

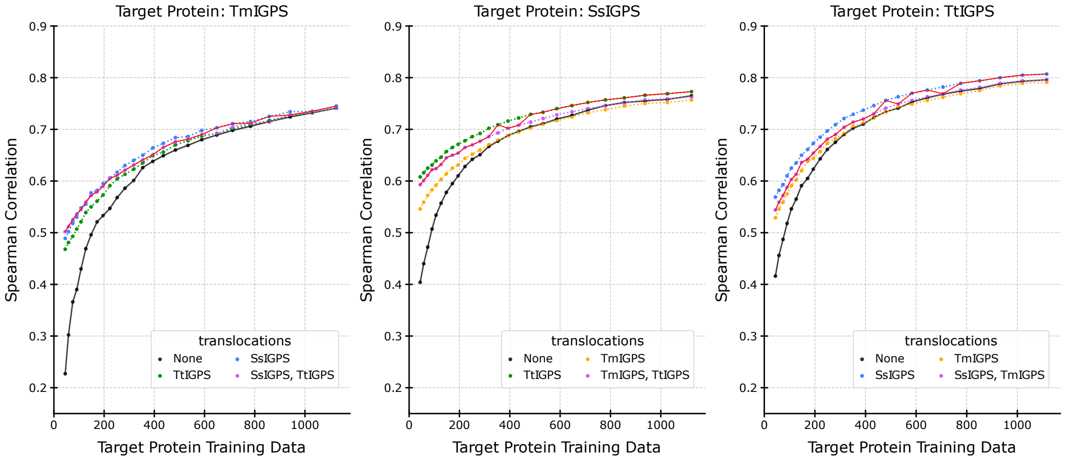

Figure S40.3: IGPS, ESM2 pLM, and SVR predictor.

Supplemental S41: IGPS, ESM2, Lasso, Statistical-Greedy - Individual-Greedy - Individual-Select

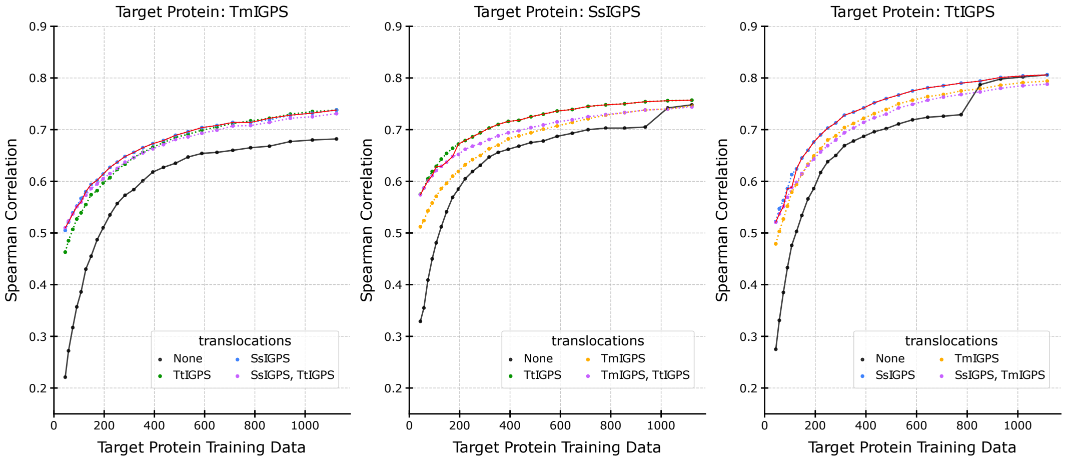

Figure S41.1: IGPS, ESM2 pLM, and Lasso predictor.

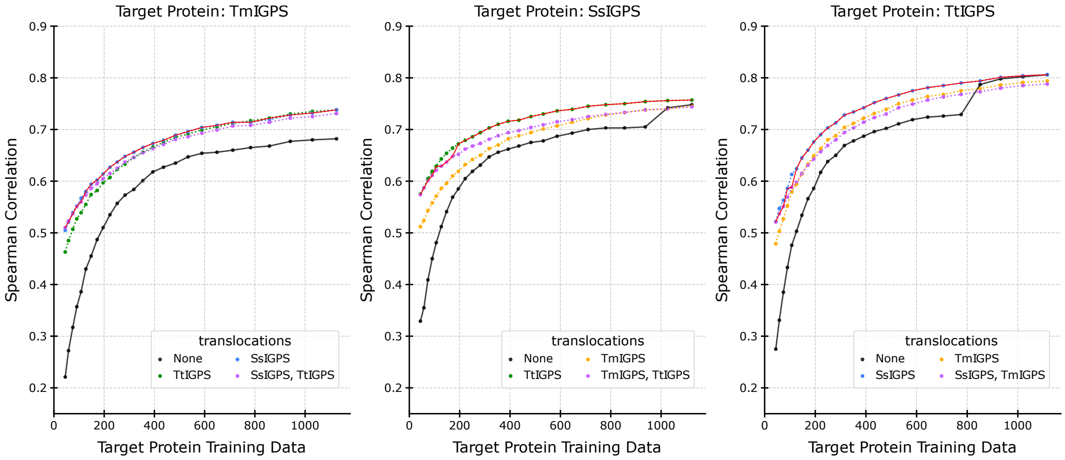

Figure S41.2: IGPS, ESM2 pLM, and Lasso predictor.

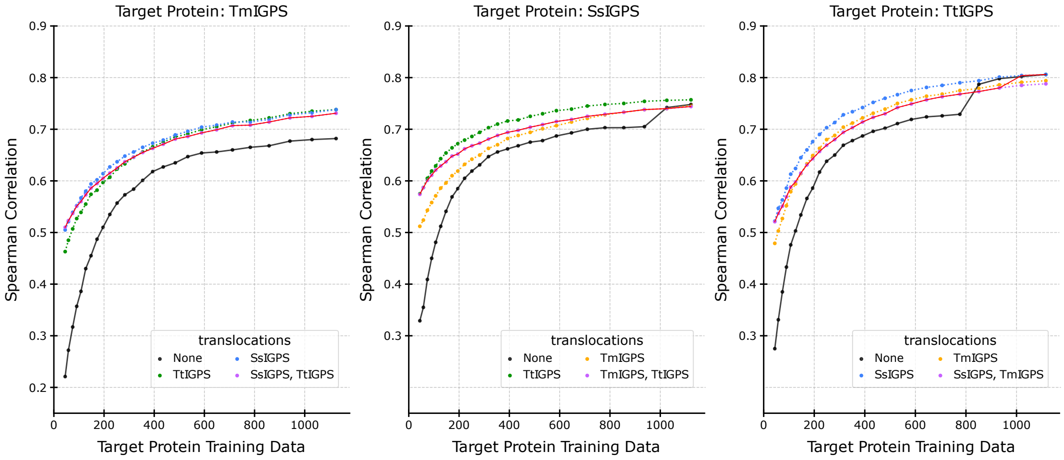

Figure S41.3: IGPS, ESM2 pLM, and Lasso predictor.

Supplemental S42: IGPS, ESM2, RF, Statistical-Greedy - Individual-Greedy - Individual-Select

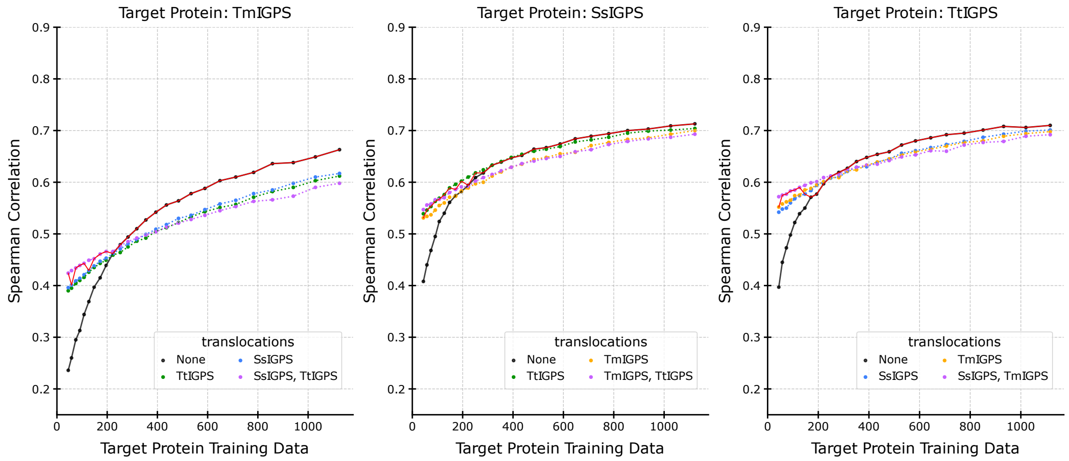

Figure S42.1: IGPS, ESM2 pLM, and RF predictor.

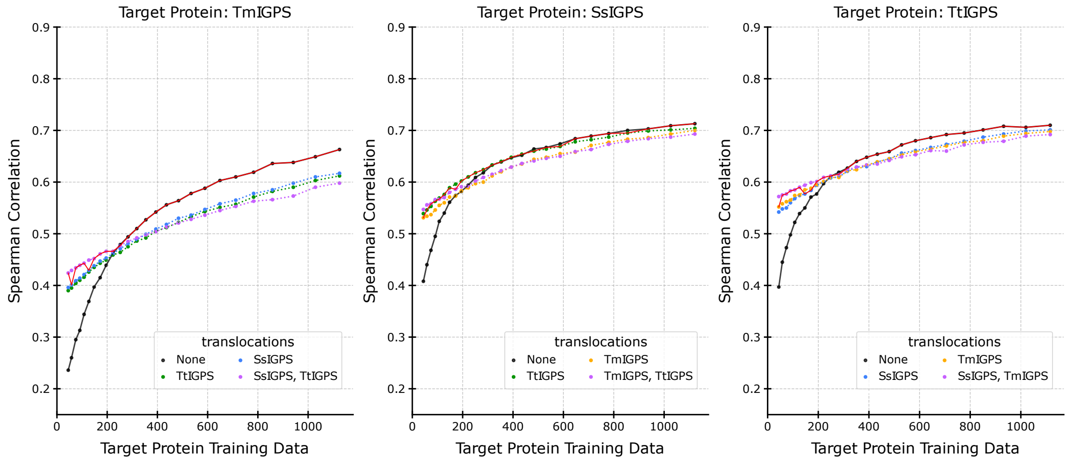

Figure S42.2: IGPS, ESM2 pLM, and RF predictor.

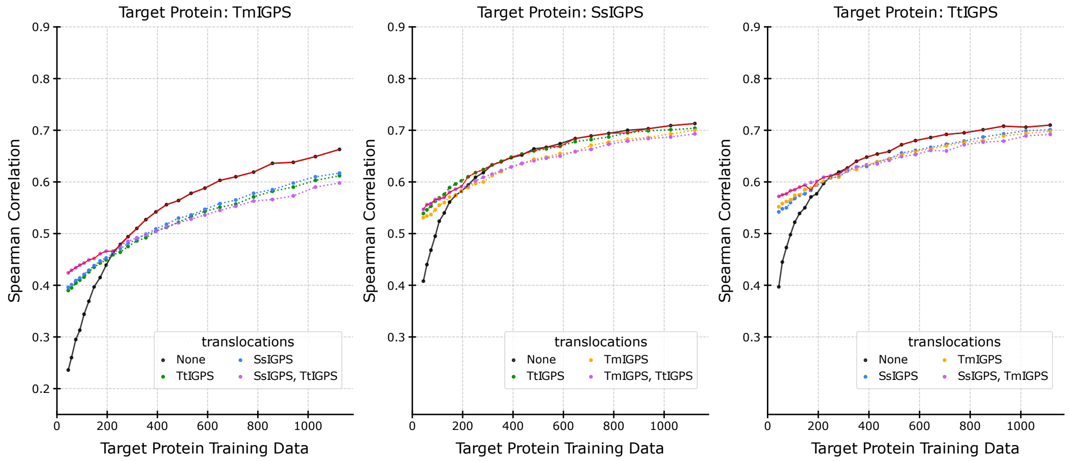

Figure S42.3: IGPS, ESM2 pLM, and RF predictor.

Supplemental S43: GFP, ESM2, SVR, Statistical-Greedy - Individual-Greedy - Individual-Select

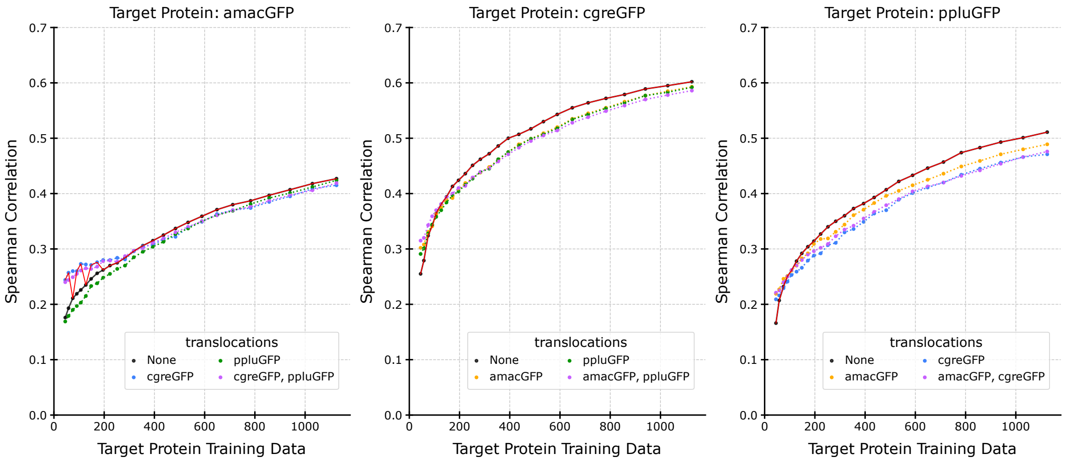

Figure S43.1: GFP, ESM2 pLM, and SVR predictor.

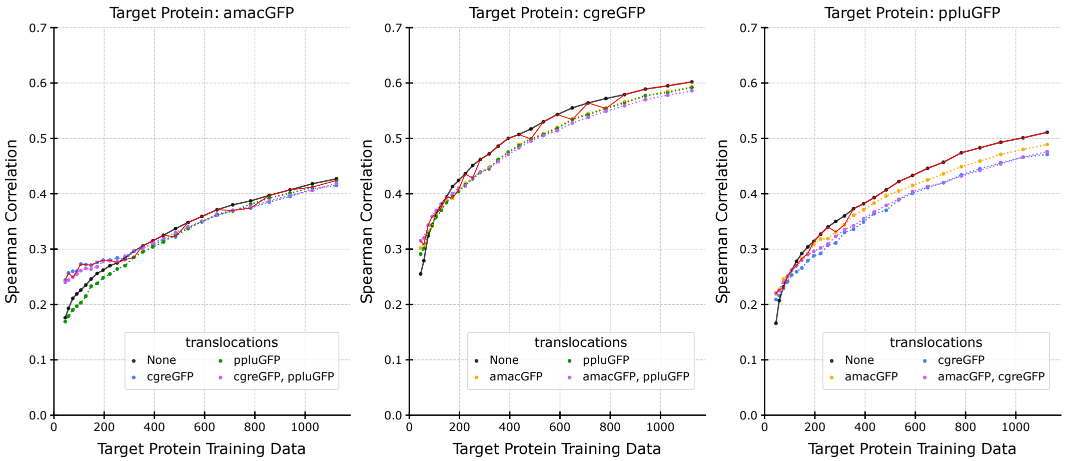

Figure S43.2: GFP, ESM2 pLM, and SVR predictor.

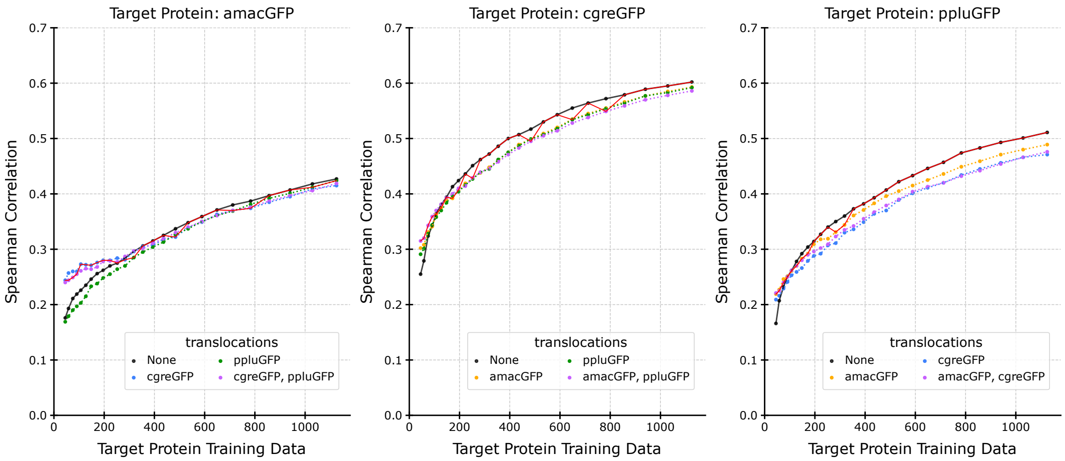

Figure S43.3: GFP, ESM2 pLM, and SVR predictor.

Supplemental S44: GFP, ESM2, Lasso, Statistical-Greedy - Individual-Greedy - Individual-Select

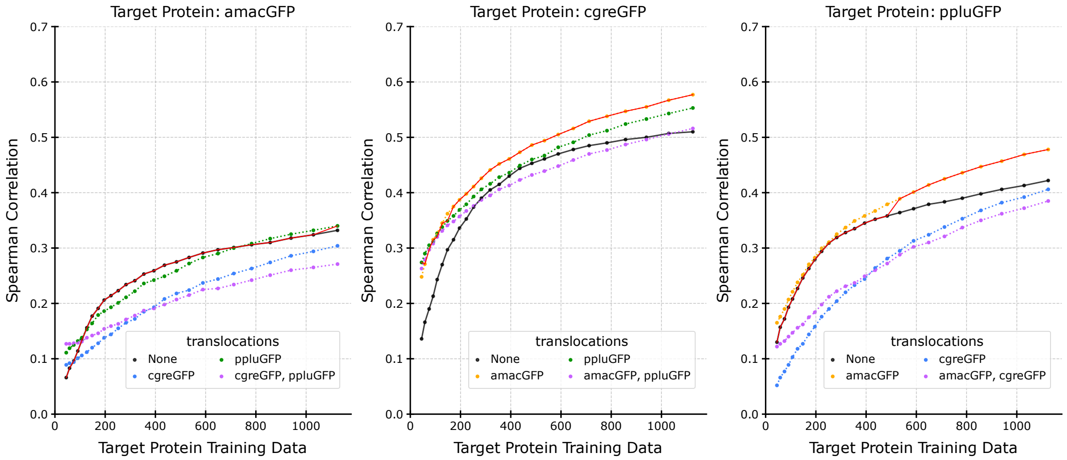

Figure S44.1: GFP, ESM2 pLM, and Lasso predictor.

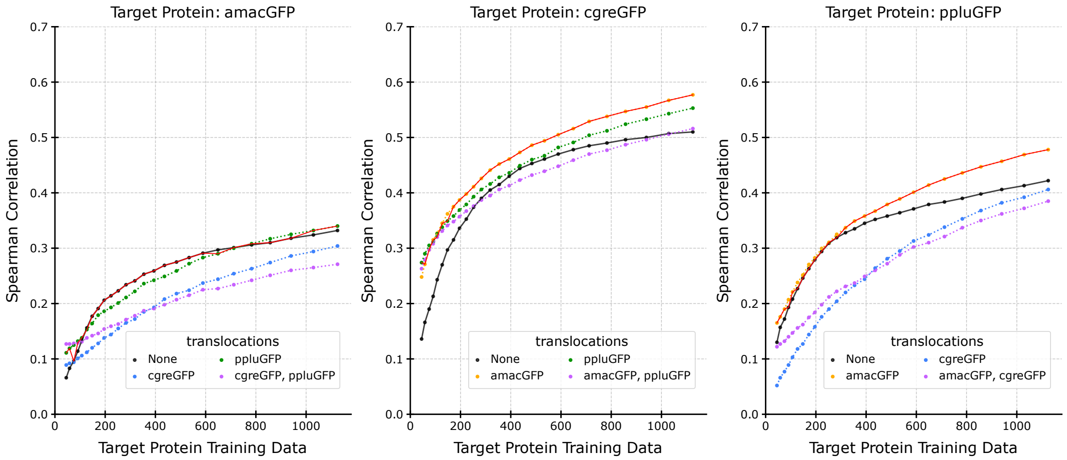

Figure S44.2: GFP, ESM2 pLM, and Lasso predictor.

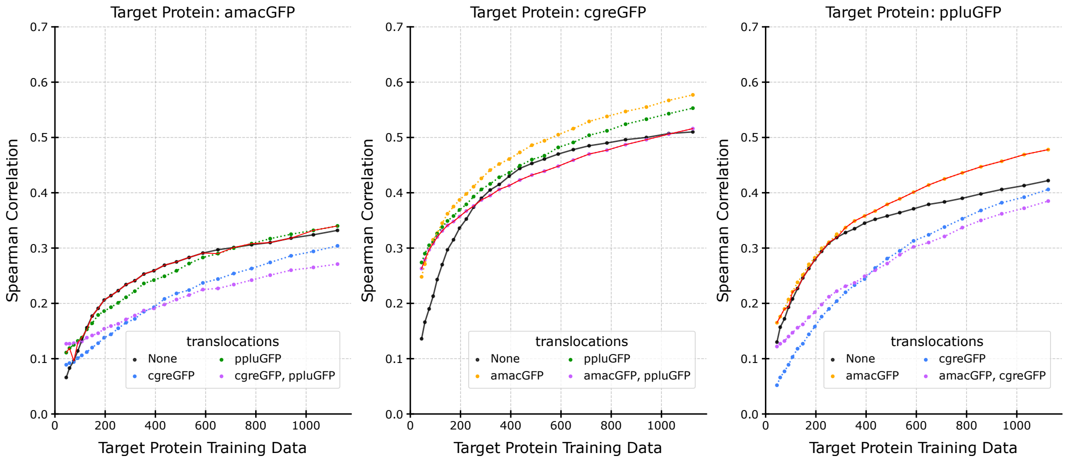

Figure S44.3: GFP, ESM2 pLM, and Lasso predictor.

Supplemental S45: GFP, ESM2, RF, Statistical-Greedy - Individual-Greedy - Individual-Select

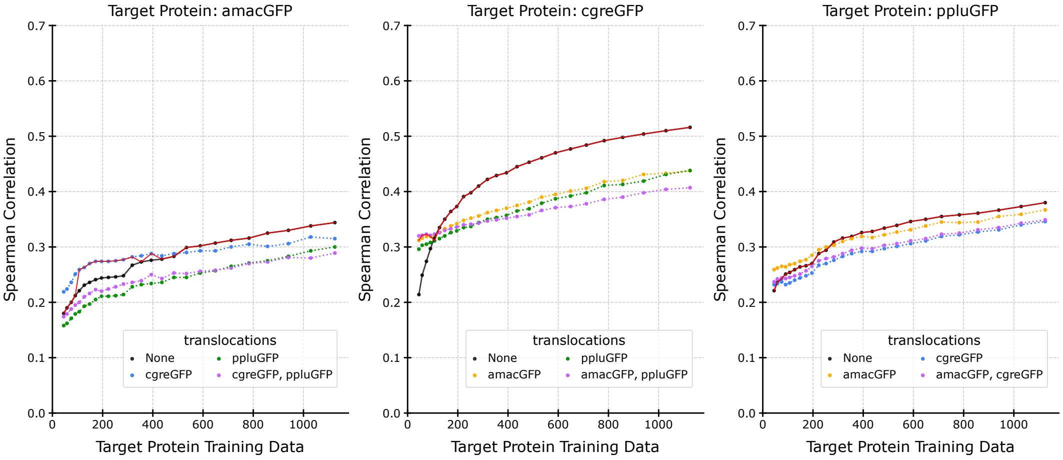

Figure S45.1: GFP, ESM2 pLM, and RF predictor.

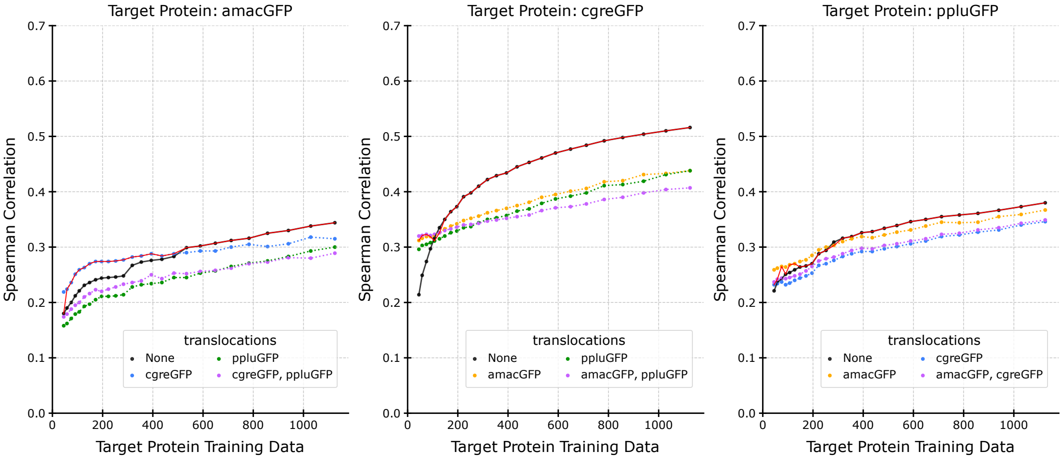

Figure S45.2: GFP, ESM2 pLM, and RF predictor.

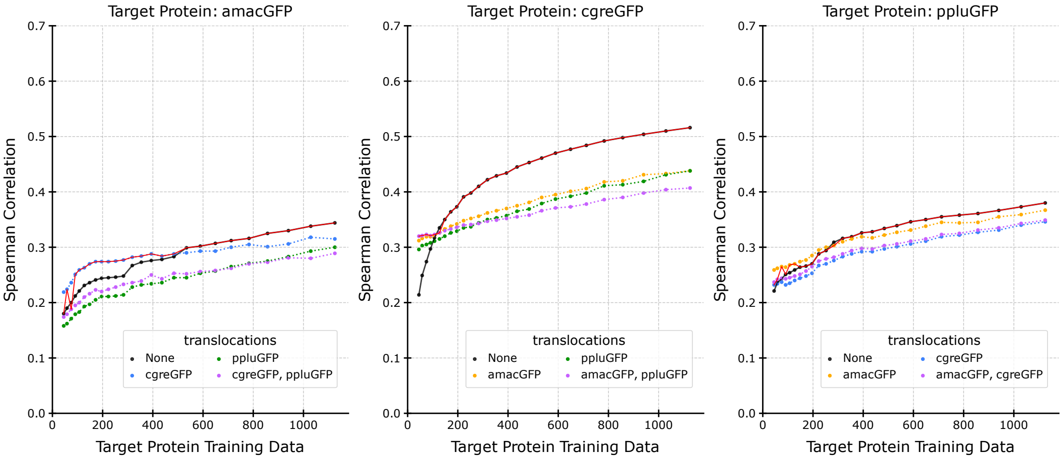

Figure S45.3: GFP, ESM2 pLM, and RF predictor.
